# Probing Prediction‐Related Processes in Language Using an EEG Word Stem Completion Paradigm

**DOI:** 10.1111/psyp.70339

**Published:** 2026-06-25

**Authors:** Hui‐Sun Chiu, Ryan J. Hubbard, Kara D. Federmeier

**Affiliations:** ^1^ Department of Psychology University of Illinois Champaign Illinois USA; ^2^ Department of Psychology State University of New York at Albany Albany New York USA; ^3^ Program in Neuroscience University of Illinois Champaign Illinois USA; ^4^ The Beckman Institute for Advanced Science and Technology Champaign Illinois USA

## Abstract

Humans comprehend language rapidly, with active prediction of upcoming words as a key mechanism. Studies using sentences have documented facilitations in behavior and brain activity (N400) when people encounter predictable words, and brain responses (anterior positivity) specific to prediction violations. However, it has been challenging to investigate prediction *formation* mechanisms, given the complex, incremental context provided by sentences. Here, we examined prediction by measuring EEG in a simple word stem completion task. Participants saw three initial letters (e.g., bro___) varying in constraint/entropy of possible completions and were asked to generate a word completion. They then saw a target that was either a probable completion (*brother*), an improbable completion (*bronze*), or a pseudoword (*brom). In Experiment 1, participants self‐reported whether the target matched their prediction; in Experiment 2 they made a lexical decision to the target and then typed in their prediction. Consistent with findings from sentences, N400s were reduced for probable completions, primarily driven by a greater proportion of match responses with highly facilitated N400 amplitudes. No anterior positivity was found to improbable words in Experiment 1, but it was present in Experiment 2. Given these task differences, the anterior positivity may therefore reflect engagement of processes to manage activation of lexico‐semantic information when there is competition, as from a strong prediction. Crucially, neural responses following the word stem (i.e., during prediction formation) showed a sustained centro‐posterior positivity that was graded with entropy (more negative for high‐entropy stems). This effect reveals that prediction‐formation processes are sensitive to the distribution of possible completions based on the context.

## Impact Statement

To explore how predictions are formed in language, we used a word stem completion task, in which people were given three initial letters and asked to think of a corresponding word. Event‐related brain potential responses to more and less probable completions showed patterns similar to more and less expected words in sentences. Critically, we could observe prediction formation processes in response to the presentation of the word stem cue. We observed a late centro‐posterior negativity that was graded by the entropy of the word stem, suggesting that when people generate predictions about words, multiple possible candidates are preactivated in parallel.

## 1 Introduction

How do people process words so fast? The average adult's silent reading rate of 238 words per minute equates to processing roughly four words per second (Curcic 2021). This rapid word recognition is crucial in many real‐life situations. Take driving, for example: In addition to interpreting colors and symbols on traffic signs, drivers must quickly read words that convey essential directions and hazards. Without this ability, the risk of accidents increases significantly (Boyce and Dax 1981). Beyond driving, strong reading skills support health, social connection, and educational and workplace success. Thus, the question of what cognitive and neural mechanisms permit rapid word apprehension is a crucial one.

One key factor that supports rapid reading and comprehension is the ability to use context information to predict features of likely upcoming words. Prediction in language has been argued to involve active processes, expanding language comprehension beyond the passive act of retrieving and integrating word meanings from long‐term semantic memory (Federmeier 2022; Huettig 2015; Kamide 2008; Kuperberg and Jaeger 2016). It has been characterized as an active, generative process in which contextual information is used to preactivate features of likely upcoming input, shaping subsequent word processing. Within this broader view, a range of theoretical approaches has sought to explain how predictive activation is implemented. Some accounts link prediction to the strength and efficacy of top‐down connectivity within the comprehension system (Federmeier 2007), whereas others formalize prediction in terms of cascaded activation linking comprehension and production (Dell and Chang 2014) or production‐based forward modeling and simulation (Pickering and Gambi 2018; Pickering and Garrod 2013). Despite differences across these accounts, they converge on the view that prediction involves the context‐driven activation of candidate representations prior to input. In turn, the anticipation and pre‐activation of words heavily relies on available contextual cues, which, in sentence processing, are incrementally provided by the prior context (Altmann and Kamide 1999; Van Petten and Kutas 1990). A common measure of word predictability in context is cloze probability, the percentage of participants who complete a given language segment with that word (Taylor 1953). Some – “strongly constraining” – contexts provide rich and informative cues, such that the most likely (“best”) completion has a high cloze probability. Conversely, within weakly constraining sentence frames, even the best completions may have relatively low cloze probabilities. Behavioral studies have long demonstrated that supportive contexts facilitate the processing of predictable, high cloze probability words, leading to faster and more accurate responses (Schuberth et al. 1981; Schwanenflugel and Shoben 1985; Simpson et al. 1989), and manipulating contextual constraint—and thereby the strength and specificity of the likely predictions people can make—has been a key strategy for research into prediction in language (Huettig 2015).

Due to the rapid pace of language comprehension, methods that can track processing with high temporal resolution—such as the derivation of event‐related potentials (ERPs) – have proven especially valuable for probing effects of constraint and expectancy in sentences. Expectancy effects have been well characterized on the N400, which is observed 300–500 ms after the onset of a word and is associated with access to long‐term semantic memory (see review in Kutas and Federmeier 2011). N400 amplitudes are strongly graded by cloze probability, with smaller (less negative) amplitudes as cloze probability increases (Kutas and Hillyard 1984; Wlotko and Federmeier 2012). This pattern reflects the extent to which prior context has already activated semantic features of an upcoming word, such that less additional retrieval is required when those features overlap with the input (reviewed in Federmeier 2022). Although the N400 is sensitive to cloze probability, it is not modulated by sentence constraint itself. Kutas and Hillyard (1984) found that N400 amplitudes remained similar for equally low cloze probability words, regardless of whether they were the best completions of weakly constraining sentences or alternative completions of moderately or strongly constraining ones. This indicates that N400 amplitude indexes the degree of alignment between contextually activated information and the incoming word, rather than the strength of an unfulfilled expectation for some other item.

Some N400 patterns in particular, such as those seen in what has been termed the “related anomaly paradigm,” attest to the fact that information about likely upcoming words can be predictively preactivated. For example, Federmeier and Kutas (1999) provided evidence that semantic features of likely upcoming words are preactivated during sentence processing, and since then, a large literature has used the N400 to examine context‐based expectancy for words as a function of orthographic, phonological, morphosyntactic, and semantic features of various kinds (Laszlo and Federmeier 2009; Urbach et al. 2020; Van Berkum et al. 2005; Wicha et al. 2004).

N400 amplitudes are thus sensitive to the match between contextually activated/predicted information and that evoked by the actual input. Other aspects of the ERP signal, instead, reveal how the system responds to prediction violations. In particular, plausible but unexpected words completing strongly constraining sentences elicit more positive responses over frontal scalp regions in a time window after the N400, between about 600 and 1000 ms post‐stimulus‐onset (Brothers et al. 2015; Federmeier et al. 2007; Hubbard and Federmeier 2021; Lai et al. 2024). This post‐N400 anterior positivity thus seems to index the greater degree of prediction violation that arises due to the presence of richer and more strongly activated semantic information predicted from a strongly constraining sentence context. Importantly, this effect is not observed when unexpected words are implausible or anomalous; such inputs instead elicit a posterior positivity in the same time window (Brothers et al. 2020; Delong et al. 2011; Kuperberg et al. 2020; Van Petten and Luka 2012). Thus, the anterior positivity is not a general response to unexpected information, but indexes processing elicited by prediction violations that can still be plausibly linked to the context.

N400 and anterior positivity effects reflect the benefits of accurate predictions and the consequences of incorrect expectations, respectively, demonstrating that the strength and content of predictions have a significant impact on on‐line word processing. However, these effects index *outcomes* of having made a prediction. They do not directly illuminate the formation of the prediction itself, which has to be built over the course of processing the sentence at multiple levels of analysis. Predictability is influenced by both local and global cues, such as semantic priming from nearby words and the retrieval of broader knowledge structures, and evidence suggests these local and global cues operate in parallel (Kutas 1993; Van Petten 1993). Predictions are also flexible and can change rapidly. Szewczyk et al. (2022) showed that an adjective before a sentence‐final target word can either strengthen the prediction for the most probable sentence completion or move predictions toward a lower probability alternative (e.g., “At night the old woman locked the ‘*front’ door/ ‘frosty’ window*.”), as revealed by N400 patterns. Because sentence constraint arises from multiple sources and prediction formation is continuous rather than tied to a single word, isolating its precise mechanisms remains a challenge.

To better probe the prediction formation process at the level of an individual word and assess the impact of constraint, the present study was designed to examine pre‐activation at a single‐word level. Our design builds on prior research investigating how sublexical cues with varying specificity influence word recognition. For example, in Central Swedish, pitch accents distinguish different words, and these tonal patterns vary in the number of possible word continuations. In general, accent 1 (characterized by a lower onset tone) allows fewer potential word continuations than accent 2 (characterized by a higher onset tone). A number of studies have identified what has come to be known as the Pre‐Activation Negativity (PrAN)—a negativity with a left fronto‐central maximum observed around 200 ms after word onset—that is larger for words that begin with more constraining (low) tones (Roll et al. 2010, 2013, 2015). Notably, in Southern Swedish, where pitch‐accent constraints are reversed, the PrAN effect shows the same relationship to constraint, ruling out acoustic explanations for the effect pattern (Roll 2015). Thus, this increased negativity has been interpreted as reflecting the level of predictive certainty.

Similar effects have also been found in other languages, albeit with varying polarity. For instance, in Danish, stød, a suprasegmental unit similar to a glottal stop, not only distinguishes between different words but also serves as a predictive cue for morphological suffixes. An increased (right) anterior negativity (280–430 ms) was observed for words with stød, which corpus data reveal leads to significantly fewer potential continuations compared to non‐stød words (Hjortdal et al. 2022). A study in English, looking at responses to auditory words with different numbers of onset competitors also found a left anteriorly‐distributed effect between 150 and 400 ms, but, in this case, of opposite polarity: increased negativity for words with *more* competitors (i.e., when the onset cue was less constraining; Söderström and Cutler 2023). Although the factors determining the precise timing, distribution, and polarity of these effects remain to be elucidated, as a set these studies show that initial sublexical cues within a single word impose constraints that influence word processing.

In the studies just described, the target words were embedded within a sentence to examine constraint effects as comprehension unfolds (and, thus, in a context wherein comprehenders might naturally be making predictions), but they provide inspiration for exploring constraint effects based on simpler properties of single words (León‐Cabrera et al. 2024). In particular, when predictions can be made based on the partial properties of single words and when the precise timing of those cues is known and can be controlled, then one can measure prediction formation directly, rather than infer it through later responses like the N400 or anterior positivity. Here, therefore, we developed a novel paradigm for examining prediction with visual word presentation, using a word stem completion task.

Although in reading, single words do not unfold over time as they do in the auditory modality, partial word cues can still be used to prompt predictions or production. One well‐established method for doing this is the *word stem completion task*, wherein participants are presented with a word stem consisting of, e.g., three initial letters (e.g., bro___) and are instructed to complete it with the first word that comes to mind. This task has been widely used in implicit memory research, as completions are sensitive to activation levels in the lexicon and reveal priming effects driven by prior exposure to the words, even in patients with amnesia (Graf and Mandler 1984; Graf and Schacter 1985; Warrington and Weiskrantz 1970, 1974). Here, outside of a memory manipulation, we decided to use word stems to cue people to predict words.

Using word stems affords precise control over predictive cues outside of a sentence context. From norming data, we can quantify the constraint of the cues and determine the distribution of likely predictions, analogous to cloze probability in sentence studies. In the current experiments, each stem was paired with three types of completions: the most probable word completion (e.g., a 60% completion probability for “brother” when given “bro___”), an improbable word completion (e.g., a 1% completion probability for “bronze”), and a pseudoword (e.g., *brom). Crucially, we manipulated the predictive strength of the word stem cue. Highly constraining stems (e.g., “sch___” → “school,” 90% probability) led most participants to generate the same completion. In contrast, low‐constraint stems (e.g., “pre___”) elicited a wider range of completions (for this example, 48 distinct responses, with all but the most common under 5%). To quantify constraint, we used entropy, a measure that accounts for the distribution of all possible completions (Hale 2003). Unlike the measures of constraint that have been used in many sentence‐processing studies, which focus solely on the probability of the most likely completion, entropy takes the distribution of responses into account, such that two stems with the same top completion probability can have different entropies depending on the probability of the alternatives.

Although word stems can offer manipulations similar to those that have been successfully used to probe prediction processes in sentences, one concern is whether explicitly prompting participants to predict a word differs fundamentally from natural language comprehension processes. Work with sentences, however, has suggested that active prediction tasks yield very similar patterns to those seen in natural, passive, reading experiments. For example, Lai et al. (2024) compared ERP results under passive comprehension, wherein participants were simply instructed to read for comprehension, versus when participants were explicitly told to try to predict the sentence endings. They found similar cloze probability effects on the N400 in both task contexts, albeit with larger overall effect sizes in the active condition, possibly due to more task engagement (cf., Hubbard and Federmeier (2021) who found similar N400 patterns, with reduced overall effect size, under a dual‐task load). Notably, neither study found differences in the presence or size of the anterior positivity effect, suggesting that core prediction mechanisms operate similarly across these kinds of task conditions. Thus, it is plausible that our active word prediction task will engage prediction mechanisms comparably.

Given the previous findings, for ERPs time‐locked to the completions we expect to observe a graded N400 response associated with completion probability. Specifically, we expect there to be a reduced N400 amplitude, indicating facilitated semantic access, for words with a higher completion probability (with N400 responses being largest for pseudowords). If the anterior positivity reflects processes associated with prediction violations that can obtain even outside of a message‐level semantic representation (as in Federmeier et al. 2010), then we might observe that improbable word completions, particularly those generated from more constraining or lower‐entropy word stems, would elicit an enhanced anterior positivity following the N400 response. Critically, in response to the stem presentation, we will look for effects akin to those observed in spoken language studies, with responses graded by entropy.

## 2 Experiment 1

In the first experiment, participants were presented with a word stem and asked to silently generate one word that would complete the stem. Subsequently, a completion was shown, which could either be the most probable word, an improbable word, or a pseudoword, and participants pressed a button to indicate whether it matched the word they had initially generated. Analyses focused on two key time windows: (1) at the presentation of the word stem, to observe the prediction generation process and its influence by the constraint of the stem and (2) at the presentation of the completion, to examine how target words with high or low probability are processed in weaker and stronger contexts, akin to sentence processing paradigms such as that of Federmeier et al. (2007). If the effect patterns time‐locked to presentation of the completion resemble those seen in prior work, this may suggest that the comprehension processes engaged in the word stem paradigm align in key ways with those engaged during sentence processing. Thus, at the presentation of the completion, we expect to see N400 responses that are graded by completion probability but, with probability held constant, not by entropy. We will also examine whether improbable completions of low entropy stems elicit an anterior positivity.

### 2.1 Method

#### 2.1.1 Participants

Final data were derived from thirty participants (mean age 19 years, range: 18–25 years; 18 self‐reported as female), recruited from the subject pool of the Department of Psychology at the University of Illinois. Because we did not know what the critical entropy effect might be like, we powered for a medium effect (d=0.5), which, given typical lab variability (SD≈1.5μV for mean amplitude difference measures), corresponds to sensitivity to ~0.75 μV differences with 30 participants. Data from two additional participants were excluded due to an excessive artifact rejection rate (> 30%) in their electroencephalogram (EEG) data after artifact correction was applied. All participants were right‐handed native English speakers who met the criteria of having normal or corrected‐to‐normal vision, no exposure to languages other than English before the age of five, and no prior history of skull fracture or neurological or psychiatric disorders. They received compensation in the form of either course credit or a cash payment. The study was approved by the Institutional Review Board (IRB) at the University of Illinois, and every participant gave written, informed consent before participating in the study.

#### 2.1.2 Materials

A total of 330 word stems were created using words selected from the Merriam‐Webster (1994) English dictionary.^1^ Each word stem was chosen to ensure that it had a minimum of five possible word completions. A norming study (*N* = 103) was used to determine the number and probability of completions participants were likely to elicit in response to each stem. The norming task was administered using Qualtrics (https://www.qualtrics.com/; Qualtrics 2005), and participants were recruited from Amazon Mechanical Turk (MTurk) (https://www.mturk.com/). The set of 330 word stems was equally divided into three lists, to which participants were randomly assigned while ensuring a nearly equal number of participants for each list. Participants were instructed to fill in the first word that came to mind to complete a given word stem; we did not place further restrictions on the instruction, so participants were, for example, free to use proper nouns. Additionally, participants were encouraged to type the word that came to mind even if they were unsure of the spelling. During data cleaning, misspellings were corrected manually to the intended words if recognizable, or were removed if unidentifiable. Nonwords and incorrect word completions (e.g., providing “example” for the word stem “exp___”) were also excluded. Ultimately, the number of usable word completions for each word stem ranged from 86 to 101.

Using the completion probabilities of all possible word completions obtained in the norming, the entropy of each word stem was computed with the formula:

$$H\left(X\right)=-\sum_{i}P\left(X_{i}\right)\;\log_{2}P\left(X_{i}\right)$$

The 330 word stems were then evenly divided into five levels or bins of entropy, from lowest to highest: LowestEnt, LowEnt, MedEnt, HighEnt, and HighestEnt. Table 1 provides the descriptive statistics of entropy values and related variables, which, for each level of entropy, include the number of completions and the completion probabilities of the most commonly generated word completions, clearly showing that word stems with lower entropy values generally have fewer possible completions and that the most commonly generated word completions for these stems have a higher average completion probability.

**TABLE 1:** Entropy values and related variables of word stems at each entropy level.

	LowestEnt (M ± SD)	LowEnt (M ± SD)	MedEnt (M ± SD)	HighEnt (M ± SD)	HighestEnt (M ± SD)
Entropy	2.04 ± 0.42	2.75 ± 0.13	3.15 ± 0.11	3.54 ± 0.12	4.12 ± 0.38
Number of completions	11.26 ± 2.76	13.91 ± 2.39	16.53 ± 3.25	19.82 ± 2.69	28.18 ± 7.12
Most common completion probability	0.56 ± 0.14	0.36 ± 0.07	0.30 ± 0.06	0.23 ± 0.06	0.17 ± 0.04

*Note:* M and SD stand for mean and standard deviation, respectively. In the last row, the most common completion probability gives the average probability of the most commonly generated word for each word stem.

Two different word completions were chosen for each word stem from the pool of word completions provided in the norming study. One was the word with the highest completion probability for that stem and the other was one of the words with the lowest completion probability for that stem, often provided by only one person. Among candidate low probability words, we excluded any that were morphological variants of or that overlapped in form with the most probable completion (e.g., “found” was the completion with the highest probability for the word stem “fou___”, and both “foundation” and “founded” were therefore excluded as possibilities for the low probability completion). Additionally, we excluded any words that might induce emotional arousal, such as profanity, insults, or sexually explicit language. Within those constraints, we selected words with the aim of reducing differences between the completion types in word length and frequency. However, there were still significant differences across lexical variables between the most probable and improbable word completions, as people were more likely to generate words that are shorter, more frequent, familiar, concrete, and easily imaginable. Table 2 provides descriptive statistics for all lexical variables, sourced from the MRC Psycholinguistic Database (https://websites.psychology.uwa.edu.au/school/mrcdatabase/uwa_mrc.htm; Wilson 1988) and the CLEARPOND Database (https://clearpond.northwestern.edu/index.php; Marian et al. 2012).

**TABLE 2:** Lexical variables for the most probable and improbable word completions in Experiment 1.

	MostProb (M ± SD)	ImProb (M ± SD)	Paired *t*‐test(*t*; *p*)
Number of letters	5.51 ± 1.33	6.61 ± 1.88	−9.71; < 0.001
Frequency (per million)	242.90 ± 1012.32	13.78 ± 29.56	4.11; < 0.001
Log Frequency	1.61 ± 0.79	0.70 ± 0.63	16.08; < 0.001
Familiarity rating (100–700)	408.65 ± 249.34	148.17 ± 230.37	14.46; < 0.001
Concreteness rating (100–700)	305.75 ± 231.39	118.87 ± 205.95	11.68; < 0.001
Imageability rating (100–700)	348.75 ± 231.36	137.15 ± 216.78	12.80; < 0.001

*Note:* The degrees of freedom for the number of letters and frequency are 329. The degree of freedom for log frequency is 310 because the frequency of some word completions is 0, making the log value undefined. For familiarity, concreteness, and imageability, the degrees of freedom are 318 since some word completions were not included in the database.

For each stem, we also created a pseudoword completion using Wuggy (http://crr.ugent.be/programs‐data/wuggy; Keuleers and Brysbaert 2010). The most probable words were designated as the reference words, and pseudowords were selected in a manner that ensured that their orthographic neighborhood size and density properties closely resembled those of the reference words. Taken together, there were thus three completions for each word stem: the word completion with the highest completion probability for that stem (mean (M): 0.33; standard deviation (SD): 0.16), an improbable word completion with the lowest (or a very low) completion probability (M: 0.01; SD: 0.008), and a pseudoword. These three conditions are designated as MostProb, Improb, and Pseudo in the following text. See Appendix A for more details.

We created six lists, each comprising 330 trials, such that each stem was presented once per list and, across lists, all stems occurred with each of their completion types the same number of times. The creation of these six lists followed a two‐step procedure. We randomly grouped the 330 word stems into three sets which, within a given list, would comprise the MostProb, ImProb, and Pseudo conditions. We then rotated the completion condition across sets and built lists from all possible set combinations, yielding 6 lists total (i.e., ABC, ACB, BAC, BCA, CAB, CBA, where the order in the triad denotes Set 1, 2, and 3, and A represents MostProb, B for ImProb, and C for Pseudo condition). Lexical variables were controlled across lists within each completion condition. The 330 trials within each list were evenly divided into 10 blocks, with an equal number of items from each condition within each block. The sequence of block presentations was arranged in a random order, and the order of stimuli within a block was also randomized. Five participants were randomly assigned to each of the 6 lists.

#### 2.1.3 Procedure

After completing paperwork, which included the informed consent form, a background questionnaire, and a handedness assessment, participants were set up for EEG and then seated in a soundproof chamber, approximately 100 cm from a CRT monitor. Task and instructions were presented using PsychoPy (Peirce et al. 2019). Each of the 10 blocks of trials took around 5–6 min on average to complete, with short breaks allowed between blocks. Trials began with a fixation cross, presented for a duration ranging from 1500 to 1750 ms; timing was jittered to reduce the contribution of slow anticipatory potentials to the ERPs of interest. Participants were instructed to silently generate the first word that came to mind upon seeing a three‐letter word stem, presented for 1000 ms. Word stems were presented as three letters followed by a line, which was always the same size (to avoid biasing word length of the responses); the stem stimulus was centered on the screen using Arial font, with a font size not exceeding 10% of the screen's height. After presentation of a second fixation cross (again, with jittered duration of 1500–1750 ms), participants saw a completion for 1000 ms. This was followed by a question mark symbol, which cued participants to respond, by pressing either the P or Q key on a keyboard, as to whether the presented completion matched what they had initially generated. The assignment of yes or no response to the P or Q key was counterbalanced across participants. Participants were asked to refrain from blinking or moving their eyes during stem and completion presentation but told they could blink freely during the response interval. Participants' responses initiated the next trial.

#### 2.1.4 EEG Recording and Processing

EEG was recorded from 26 evenly spaced Ag/AgCl electrodes attached to an Electro‐Cap. The sites from the front to the back are Midline Prefrontal (MiPf), Left and Right Lateral Prefrontal (LLPf and RLPf), Left and Right Medial Prefrontal (LMPf and RMPf), Mediolateral Frontal (LDFr and RDFr), Lateral Prefrontal (LLPf and RLPf), Medial Frontal (LMFr and RMFr), Midline Central (MiCe), Mediolateral Central (LDCe and RDCe), Medial Central (LMCe and RMCe), Midline Parietal (MiPa), Lateral Temporal (LLTe and RLTe), Mediolateral Parietal (LDPa and RDPa), Medial Occipital (LMOc and RMOc), Lateral Occipital (LLOc and RLOc), and Midline Occipital (MiOc). Furthermore, six additional electrodes were attached to the left and right mastoids, the left and right outer canthus of the eye (for detecting horizontal eye movements), and the left and right infraorbital ridge of the eye (for detecting eye blinks). The left mastoid electrode served as the online reference, while the average of the left and right mastoid electrodes was used as the offline reference for all other scalp electrodes.

Electrode impedances were kept below 5 kΩ. EEG signals were amplified using a BrainVision BrainAmp DC equipped with a 16‐bit A/D converter, an input impedance of 10 MΩ, a bandpass filter spanning 0.016–250 Hz, and a sampling rate of 1 kHz. The raw EEG data were processed using a 0.1–30 Hz second‐order Butterworth band‐pass filter with a 24 dB/oct roll‐off. Time windows spanning from 200 ms before the onset of stimulus to 1000 ms after the onset of stimulus were extracted from the continuous EEG data to epoch the EEG data, with the 200 ms pre‐stimulus time window subtracted for baseline correction.

For artifact detection and correction, a bipolar Vertical Electrooculogram (VEOG) channel was created by subtracting signals from the left infraorbital ridge of the eye from LLPf. Additionally, a bipolar Horizontal Electrooculogram (HEOG) channel was created by subtracting signals from the left and right outer canthus of the eye. Blinks were detected with an EEGLAB function measuring peak‐to‐peak activity in a moving window (200 ms, 50 ms steps), using participant‐specific thresholds (M: 95 μV; SD: 24) set through condition‐blind inspection of the data. Eye‐movement artifacts were detected using participant‐specific thresholds (M: 40 μV; SD: 10) for an EEGLAB function looking for step‐like activity in a moving window (400, 10 ms steps). If the number of blink artifacts did not exceed 10% and the total number of trials contaminated by artifacts did not exceed 25%, then contaminated trials were rejected. If blinks were detected in more than 10% of epochs, the data were subjected to an ICA decomposition algorithm (AMICA; Palmer et al. 2012) and components correlated with VEOG signals at 0.6 or more were eliminated. Similarly, we rejected trials with eye movement artifacts when fewer than 10% of the epochs were affected and used ICA correction otherwise. On average, 2 components (range 1–5) were removed. Finally, for all other scalp channels, drift and large noise was eliminated: Epochs exhibiting peak‐to‐peak activity surpassing threshold (M: 140 μV; SD: 20) were removed. On average, 6.1% (range 0%–26.5%) of epochs in each participant were rejected due to artifacts.

#### 2.1.5 Event‐Related Potentials

ERPs were measured using EEG epochs time‐locked to the onset of both word stems and completions across the five conditions of word stems (i.e., LowestEnt, LowEnt, MedEnt, HighEnt, and HighestEnt conditions) and the three completion conditions (i.e., MostProb, ImProb, and Pseudo conditions). ERPs were further filtered with a 20 Hz low‐pass filter with a 24 dB/oct roll‐off prior to measurement, and a 10 Hz low‐pass filter with a 24 dB/oct roll‐off was applied to grand averages for visualization purposes only. For comparing completion conditions, we concentrated on two temporal/spatial regions of interest (ROI), derived from the sentence processing work on which the design of the present study was based (Federmeier et al. 2007). N400 mean amplitudes were measured from 300 to 500 ms over the MiCe, LMCe, RMCe, and MiPa channels (Central ROI). The anterior positivity was measured as mean amplitude from 600 to 900 ms using a Frontal ROI encompassing the MiPf, LMPf, and RMPf channels.

For epoched EEG time‐locked to the onset of word stems (i.e., −200 to 1000 ms relative to stimulus onset, with −200 to 0 ms used for baseline correction), we used a cluster‐based permutation test (Luck and Gaspelin 2017; see also Maris and Oostenveld 2007), as there was no a priori information available to target specific time windows and regions of interest for observing entropy effects. The analysis was implemented using the FieldTrip toolbox in Matlab (Oostenveld et al. 2011). First, Pearson's *r* was calculated between the EEG data of all 330 trials and the entropy values of the 330 word stems for each time point (0–1000 ms) and across all 26 scalp electrodes, identifying significant data points with a *p*‐value lower than the critical alpha level of 0.05. Spatiotemporal adjacent clusters were then identified, and their cluster‐based statistics were computed by summing the *t* values. Next, to construct a null distribution of spatiotemporal cluster‐level statistics under the assumption of no entropy effect, a Monte Carlo cluster‐based permutation test with 2000 random permutations was conducted. For each permutation, the entropy values were randomly shuffled across trials, and the cluster with the largest sum of *t* values was considered for the permutation distribution. The observed clusters from the initial step were subsequently compared against this permutation distribution, identifying the clusters within the highest or lowest five percentiles. Finally, we analyzed the word stems as a function of entropy in the time windows and channels from these clusters.

### 2.2 Results

#### 2.2.1 Behavioral Results

Participants were instructed to press a key to indicate whether the presented completion matched their initially‐generated word. The match rate, calculated with a match response coded as one and a mismatch response coded as zero, was assessed with a 3 (Completion Condition) × 5 (Entropy) repeated measures analysis of variance (ANOVA). Results revealed main effects of completion condition $\left[F\left(2,58\right)=188.05,p<0.001\right]$ and Entropy $\left[F\left(4,116\right)=36.54,p<0.001\right]$. The pattern of match rates for the completion conditions aligned well with the norming results, as the match rate was significantly higher for the MostProb condition (M: 0.30; SD: 0.12) compared to the ImProb condition (M: 0.07; SD: 0.09) [95% CI [0.21, 0.26], $t\left(29\right)=18.46,p=<0.001$], and was very low in the Pseudo condition (M: 0.01; SD: 0.02). As shown in Table 3, there was a significant Entropy by Completion Condition interaction [$F\left(8,232\right)=39.59,p<0.001$], reflecting the fact that there was a graded decrease in match rate as entropy increased for the MostProb condition, but, in contrast, match rates did not vary systematically with entropy for the ImProb condition.

**TABLE 3:** Match rate for completion conditions at each stem entropy level in Experiment 1.

	LowestEnt (M ± SD)	LowEnt (M ± SD)	MedEnt (M ± SD)	HighEnt (M ± SD)	HighestEnt (M ± SD)
MostProb	0.51 ± 0.13	0.33 ± 0.14	0.28 ± 0.16	0.21 ± 0.16	0.18 ± 0.14
ImProb	0.07 ± 0.08	0.07 ± 0.10	0.06 ± 0.12	0.08 ± 0.10	0.06 ± 0.11

#### 2.2.2 ERP Results

##### 2.2.2.1 Word Completions

**FIGURE 1:**
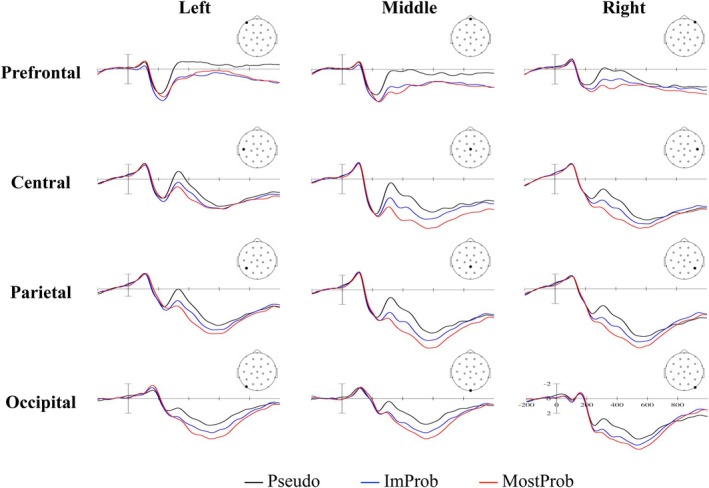
Grand average ERPs at 12 representative channels for the MostProb, ImProb, and Pseudo conditions, time‐locked to presentation of the word completion in Experiment 1. The 12 electrode sites arranged from left to right, top to bottom include LLPf, MiPf, RLPf, LDCe, MiCe, RDCe, LDPa, MiPa, RDPa, LLOc, MiOc, and RLOc. The x‐axis unit represents time in millisecond (ms), while the y‐axis unit represents voltage in microvolts (μV). Negative is plotted up in this and all subsequent figures.

Figure 1 displays grand average ERPs from all 30 participants to the three completion conditions, at a representative set of electrodes distributed across the scalp. The ERP pattern exhibits standard visual components: a negativity (N1) peaking between ~100 ms (frontal sites) and ~150 ms (posterior sites), followed by a positivity (P2) peaking between 200 and 300 ms. Following the P2, there is a negativity (N400), largest over medial centro‐parietal scalp regions, which shows a graded response to word probability.

##### 2.2.2.2 N400 Effects (300–500 ms, Central ROI)

**FIGURE 2:**
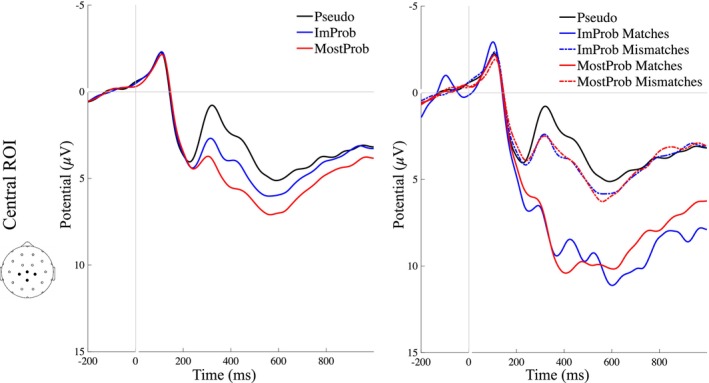
Grand average ERPs over the Central ROI to completion conditions in Experiment 1. The left side of the figure shows the three experimental conditions (MostProb, ImProb, and Pseudo). On the right side of the figure, the MostProb and ImProb conditions are further broken down by whether participants categorized the item as a match or mismatch.

Figure 2 shows ERPs averaged across channels in the Central ROI used to measure the N400. The left graph depicts the N400 pattern with word expectancy, which reveals the smallest (least negative) N400 for the MostProb condition, a larger N400 for the ImProb condition, and the largest N400 for the Pseudo condition. Planned comparisons indicated that N400 amplitude for the MostProb condition (M: 6.06 μV; SD: 4.87; standard error (SE): 0.89) was significantly lower (less negative) than that for the ImProb condition (M: 4.35 μV; SD: 4.81; SE: 0.88) [95% CI [1.07, 2.36], $t\left(29\right)=5.44,p<0.001$], and, in turn, N400 amplitude for the ImProb condition was significantly lower than that for the Pseudo condition (M: 2.25 μV; SD: 4.59; SE: 0.84) [95% CI [1.42, 2.78], $t\left(29\right)=6.30,p<0.001$]. Thus, we observe a pattern of N400 sensitivity to probability similar to the well‐characterized pattern in sentences.

N400 responses for word completions in both the MostProb and ImProb conditions consisted of a mixture of trials in which presented words either matched or did not match what participants generated in response to the stem. As shown in the analysis of behavioral responses, the MostProb condition contained more match trials, with around 30% of responses matching, compared to only 7% for the ImProb condition. Thus, N400 amplitude differences to the conditions might be driven solely or primarily by trials on which the presented word matched the participant's expectations. To examine this, ERPs for word completions were binned based on participants' match responses. The right graph in Figure 2 illustrates the N400 probability effect sorted based on participants' reported match or mismatch responses. For statistical analysis, to account for the substantial differences in the number of match trials across the MostProb and ImProb conditions (the Pseudo condition was not used in this analysis, as there were essentially no match responses in this case), a linear mixed‐effects model analysis was used with single‐trial data. The analysis was conducted using R with the lme4 package (Bates et al. 2015), with mean amplitudes within the 300–500 ms time window in the Central ROI as the dependent variable. The fixed effects included word completion condition (only MostProb and ImProb, two levels) and match responses (Matches or Mismatches), both coded using contrast coding (1, −1). This coding scheme was also applied in all subsequent analyses. Random effects included participants and stems. The model specification was as follows: N400 ~ 1 + Condition × Matches + (1|Participant) + (1|Stem). The model outcome revealed only a significant main effect of match responses [b=2.95,SE=0.21,t=13.86,p<0.001], with smaller N400 to Matches than to Mismatches. There was no significant main effect of condition [b=0.35,SE=0.21,t=1.71,p=0.09] and no interaction effect [b=0.29,SE=0.21,t=1.40,p=0.16]. Thus, condition‐based N400 differences seem to primarily reflect differential proportions of expectancy matches, which yielded much more positive ERP responses.

**FIGURE 3:**
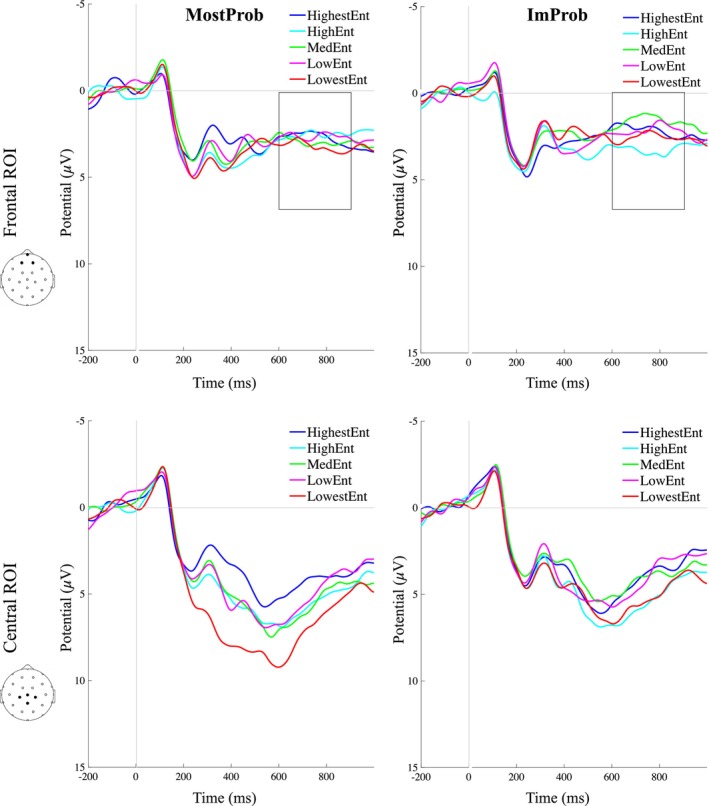
Grand average ERPs over the Frontal ROI (top row) and the Central ROI (bottom row) to entropy conditions (including both match and mismatch trials) in Experiment 1. The 600–900 ms time window used to analyze the anterior positivity is marked with a rectangle on the frontal region plots.

We also examined whether entropy affected N400 responses to the target words, in order to see if we obtain patterns similar to those previously characterized for sentential constraint. Figure 3 shows ERP responses across five entropy levels, separately for the MostProb and ImProb conditions. In the case of the MostProb condition (left side of Figure 3), where entropy and match rates are confounded, N400 amplitudes (bottom row for Central ROI) appear to decrease with lower entropy, reflecting the differential proportion of match responses across entropy condition. In contrast, for the ImProb condition (right side of Figure 3), where, critically, entropy effects can be examined with match rates held constant, there was no clear pattern associated with entropy. Entropy effects on the N400 were analyzed using a linear mixed‐effects model conducted with single‐trial data. The fixed effects included a categorical variable representing word completion conditions (only MostProb and ImProb, two levels), a continuous variable representing entropy values, and random effects for participants and stems. The model specification for the lmer() function was as follows: N400 ~ 1 + Condition × Entropy + (1|Participant) + (1|Stem). The analysis revealed a significant interaction effect between condition and entropy [b=−0.71,SE=0.17,t=−4.16,p<0.001], along with a significant main effect of condition [b=3.07,SE=0.54, t=5.63,p<0.001] and a significant main effect of entropy $\left[b=-0.83,\mathrm{SE}=0.19,t=-4.36,p<0.001\right]$. To follow up on these effects, separate linear mixed‐effects analyses were conducted for the MostProb and ImProb conditions separately, with entropy as the fixed effect. The main effect of entropy was significant only for the MostProb condition [b=−1.53,SE=0.26,t=−5.97,p<0.001]. Entropy did not reliably affect N400 amplitudes in the ImProb condition [b=−0.11,SE=0.27,t=−0.43,p=0.67]. Thus, we replicate sentence processing work showing that entropy (constraint) does not influence N400 when match rate (cloze probability) is held constant.

##### 2.2.2.3 Anterior Positivity Effects (600–900 ms, Frontal ROI)

Based on patterns observed in sentences, we probed for a post‐N400 anterior positivity effect sensitive to entropy. If responses in the word stem task replicated patterns seen in sentence processing, we expected that in the ImProb condition (right side of Figure 3), we might observe larger positivity (top row for Frontal ROI) in the lower entropy condition (where a stronger prediction would have been violated). However, there was no clear pattern of entropy effects in this condition. A linear mixed‐effects model was conducted to analyze the mean amplitudes in the Frontal ROI within the time frame of 600–900 ms. This model included a categorical variable representing word completion conditions (only MostProb and ImProb, two levels) and a continuous variable representing entropy values, with participants and stems as the random effects. The model specification for the lmer() function was as follows: Anterior. Positivity ~1 + Condition × Entropy + (1|Participant) + (1|Stem). The model yielded a significant main effect of condition $\left[b=1.24,\mathrm{SE}=0.62,t=2.02,p<0.05\right]$, but not a significant main effect of entropy [b=−0.32,SE=0.20,t=−1.59,p=0.11], nor a significant interaction effect between condition and entropy [b=−0.31,SE=0.19,t=−1.61,p=0.11]. Thus, there was no evidence for an anterior positivity effect sensitive to entropy in Experiment 1.

#### 2.2.3 Word Stems

**FIGURE 4:**
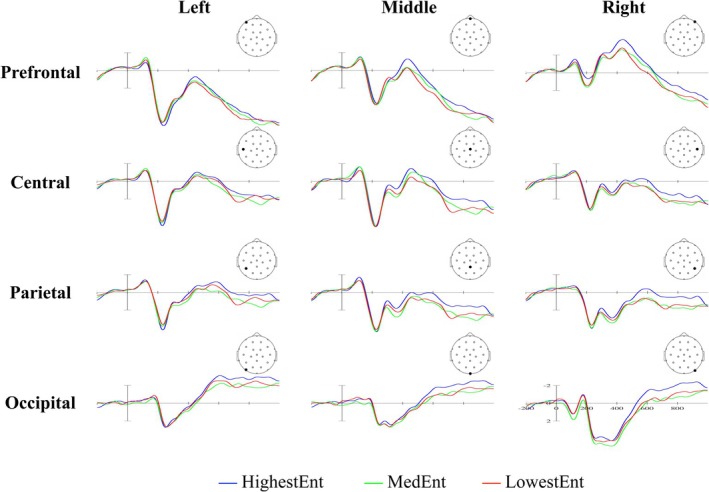
Grand average ERPs at 12 representative channels for three binned entropy levels (highest, medium, lowest), time‐locked to presentation of the word stem in Experiment 1.

Figure 4 displays grand average ERPs from all 30 participants time‐locked to the presentation of the word stems. Again, there are typical visual components, including the visual N1 (100–150 ms peak) followed by the P2. Over much of the head, these are followed by a negativity (N400), peaking around 400 ms. Over occipital sites, there is an asymmetric response following the P2, with more positivity over RLOc (contralateral to the half of the stem containing the letters) compared to LLOc. Notably, then, there is a sustained posterior negativity, beginning around 600 ms that shows a graded effect of entropy, with enhanced negativity for word stems with higher entropy values.

##### 2.2.3.1 Cluster‐Based Permutation Tests

**FIGURE 5:**
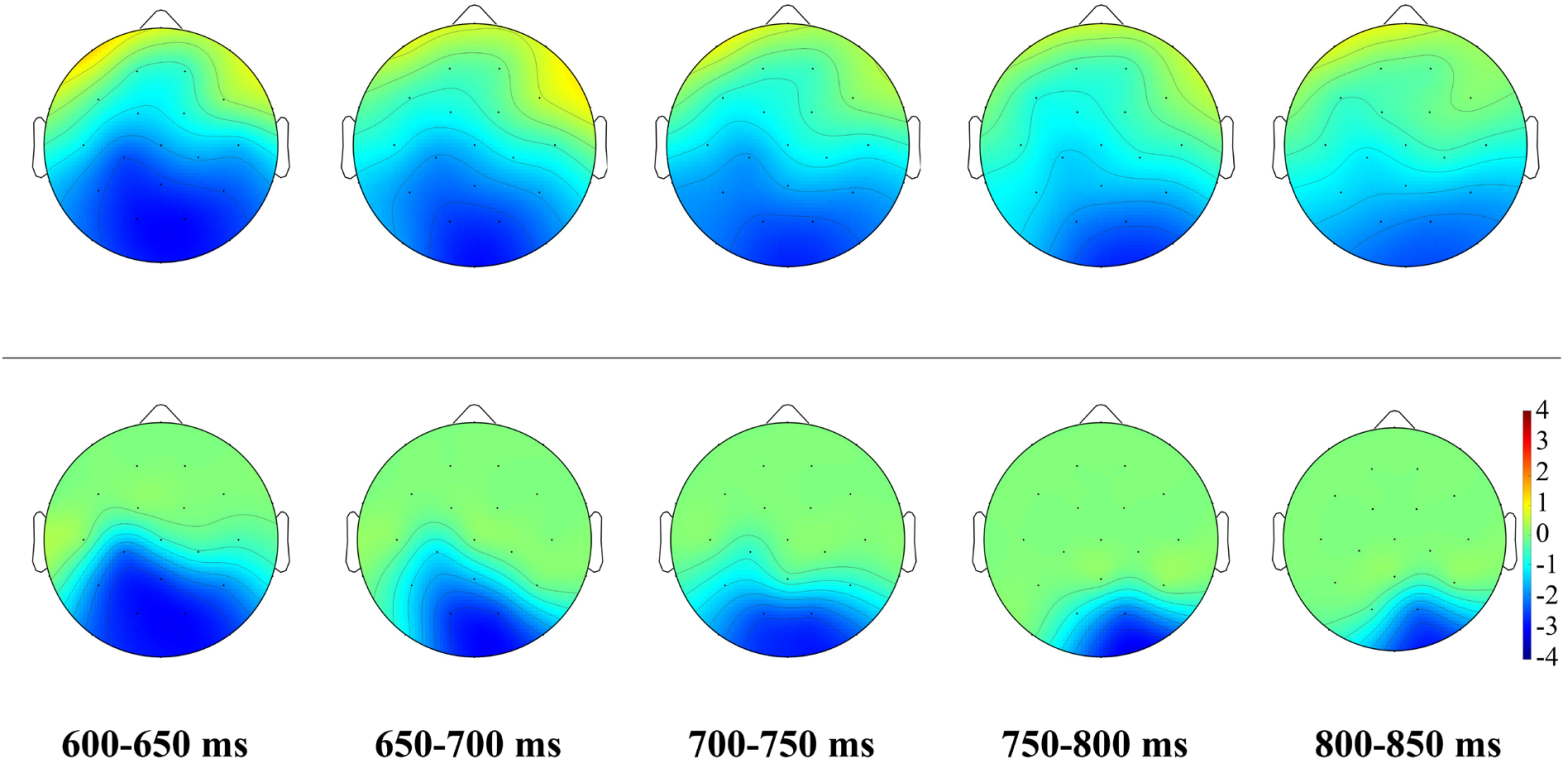
Temporal evolution of the correlation between EEG and entropy. The top row displays topographic plots of the raw results without any masks. The bottom row shows the data masked by the only cluster that passed the threshold of a Monte Carlo *p*‐value less than 0.05. The color bar represents the *t*‐value averaged over the time window.

Five clusters were identified consisting of spatiotemporally adjacent samples showing a significant $\left(p<0.05\right)$ correlation between EEG data and entropy. One of these showed a Monte Carlo *p*‐value less than 0.05 [$t=-3837.34\;\left(\mathrm{sum}\;\text{of}\;t-\text{values in the cluster}\right),p<0.05$]. This cluster indicates a significant negative correlation between entropy and the EEG signal occurring from 593 to 849 ms after stimulus onset. Figure 5 shows the topography of the cluster between 600 to 850 ms. The electrodes within the cluster include those in the central and posterior regions: LMCe, RMCe, RDCe, MiCe, MiPa, LLTe, RLTe, LDPa, RDPa, LLOc, RLOc, LMOc, RMOc, and MiOc.

##### 2.2.3.2 Centro‐Posterior Negativity (600–850 ms)

**FIGURE 6:**
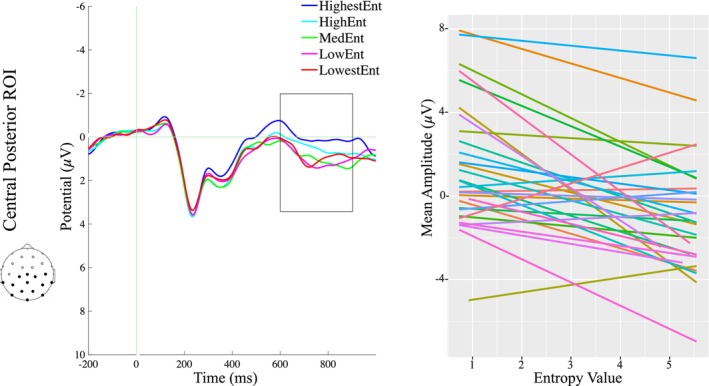
Effects of entropy at the word stem in Experiment 1. The left side of the figure shows grand average ERPs over the central and posterior regions across five binned levels of entropy. The black rectangle shows the critical time window from 600 to 850 ms. The right side of the figure plots the effect of entropy for individual participants.

Figure 6 shows ERP results averaged across channels within the significant cluster identified in the cluster‐based permutation test, while the right line plot displays the effect of entropy for each participant, both revealing an enhanced negativity with higher entropy levels. Effects were analyzed with a linear mixed‐effects model with entropy as a continuous fixed effect and participants and stems as the random effects. The model was specified as follows: Centro‐Posterior. Negativity ~1 + Entropy + (1|Participant) + (1|Stem). The analysis yielded a significant main effect of entropy [b=−0.49,SE=0.12,t=−4.07,p<0.001].

### 2.3 Interim Summary

The word completion condition manipulations were successful, as the match rate was notably higher for the MostProb compared to the ImProb word completions and increased with decreasing stem entropy. Examination of the ERP responses to the word completions revealed an N400 effect graded with word probability, similar to effects of cloze probability in sentences. Specifically, MostProb word completions were graded by probability and exhibited reduced N400 amplitudes in comparison to ImProb completions overall, but N400s to ImProb completions were not affected by entropy. Examining responses sorted by participants' match responses revealed that N400 facilitations were strongly driven by whether or not the completion matched what the participant had generated, with no additional effects of condition or entropy. This phenomenon is similar to previous findings by Lai et al. (2024), where the N400 was sensitive to the alignment between sentence endings and participants' predictions and was not further affected by the constraint of the sentence context. Different from patterns seen in sentence processing, there was no evidence for an anterior positivity effect to prediction violations.

Importantly, ERPs to the word stems revealed an effect associated with prediction formation, in the form of a negativity observed between about 600 and 850 ms over central and posterior sites, which increased with increasing entropy levels. This effect was thus functionally similar to PrAN‐like effects, in showing a sensitivity to contextual constraint based on partial word input. However, it had a different (later) timecourse and distribution (centro‐posterior rather than fronto‐central) and a polarity similar to past English (but not Swedish/Danish) studies, as less predictive cues yielded more negativity over medial posterior regions (Söderström and Cutler 2023). Given the novelty of this effect, in Experiment 2 we set out to replicate it and to determine whether it remains robust in the context of different stimuli and altered task demands. Since match trials were relatively infrequent (approximately 19% across all trials), we modified the task demands during completion presentation to reduce the likelihood of eliciting additional target matching responses such as the P3b component (Polich 2007). We also changed the task so that we could obtain specific information about participants' predictions and thereby sort ERP responses based on different dimensions of similarity between their predictions and the targets we presented.

## 3 Experiment 2

In Experiment 2, we aim to replicate and extend the findings of Experiment 1 using the same word stem completion task but with several key adjustments. We adjusted the ImProb condition words to better match the MostProb completions on lexical properties (although complete control was not possible). We also changed the task, so that participants made a lexical decision response to the presented completions, allowing us to examine ERPs as a function of completion predictability when participants were not explicitly judging match to their predictions. Instead, to identify and (later) bin match and mismatch trials, as well as to have more precise information about participants' expectations, we asked participants to type out the word they had generated after completion of the lexical decision task.

In Experiment 2, we allowed participants to self‐pace between cue and target presentation, ensuring that they had enough time to come up with a completion and that we could measure how long that took. The task sequence in Experiment 2, therefore, has participants generating a completion, pressing a button to initiate the lexical decision task, responding to the lexical decision task, and then typing in the completion they generated. Finally, a recognition task was added at the end of the experiment to probe for downstream effects of expectancy and constraint on the information participants retained about the words in the experiment. Participants were not informed in advance about the memory task, so that it would not affect their performance during the main task.

We anticipate observing a similar N400 effect pattern in response to the presentation of the completion (here for the lexical decision task). We will again probe for an anterior positivity to see if changing the task causes that effect to emerge. We also predict that we will replicate the entropy‐sensitive negativity elicited by the word stem presentation. These effects, if obtained despite the adjustments made in Experiment 2, would support the idea that the negativity observed at the cue reflects the prediction formation process, which is sensitive to constraint, and that the resulting predictions affect subsequent word processing.

### 3.1 Methods

#### 3.1.1 Participants

Thirty participants (mean age 20 years, range: 18–28 years; 16 self‐reported as female) were recruited from the subject pool system of the Department of Psychology at the University of Illinois, gave written, informed consent, and were compensated with course credit or cash payment. Experiment 2 used the same target sample size as Experiment 1 (30 participants), which provided sufficient statistical power to detect the effects of interest, to maintain consistency and allow for direct comparison across experiments. Data from five additional participants were excluded because of excessive artifacts (> 30%) in their EEG data. None of them had previously participated in Experiment 1. The eligibility criteria were the same as Experiment 1.

#### 3.1.2 Materials

We used the same 330 word stems from Experiment 1. The completions in both the MostProb and Pseudo conditions remained unchanged from Experiment 1. However, we modified the ImProb condition, resulting in 64% of these items being different in Experiment 2. Our aim was to try to reduce the lexical differences between the MostProb and ImProb condition, as well as to ensure that findings were robust across the choice of low probability items. ImProb items were still chosen from the set of very low probability completions (generated by 5 or fewer people). Although we were not able to fully eliminate lexical differences between the MostProb and ImProb word completions, compared to Experiment 1 the ImProb word completions in Experiment 2 were more similar to the MostProb ones in being shorter [95% CI[−0.52, −0.89], *t*(329) = −7.53, *p* < 0.001], and more frequent [95% CI[9.36, 25.66], *t*(329) = 4.23, *p* < 0.001], familiar, [95% CI[66.64, 127.16], *t*(317) = 6.30, *p* < 0.001], concrete [95% CI[42.99, 97.58], *t*(317) = 5.07, *p* < 0.001], and imageable [95% CI[51.46, 107.94], *t*(317) = 5.55, *p* < 0.001]; details in Table 4 and Appendix A.

**TABLE 4:** Lexical variables of the most probable and improbable word completions in Experiment 2.

	MostProb (M ± SD)	ImProb (new) (M ± SD)	Paired *t*‐test (*t; p*)
Number of letters	5.51 ± 1.33	5.90 ± 1.40	−4.32; < 0.001
Frequency (per million)	242.90 ± 1012.32	31.29 ± 76.08	3.90; < 0.001
Log Frequency	1.61 ± 0.79	1.04 ± 0.67	10.18; < 0.001
Familiarity rating (100–700)	408.65 ± 249.34	241.89 ± 261.60	8.51; < 0.001
Concreteness rating (100–700)	305.75 ± 231.39	188.56 ± 235.06	7.00; < 0.001
Imageability rating (100–700)	348.75 ± 231.36	214.75 ± 239.86	7.75; < 0.001

*Note:* The MostProb word completions are the same as those in Experiment 1, whereas the ImProb word completions had around 64% difference compared to Experiment 1. Similar to Experiment 1, the degrees of freedom for the number of letters and frequency are 329. The degrees of freedom for log frequency is 308 because the frequency of some word completions is 0, making the log value undefined. For familiarity, concreteness, and imageability, the degrees of freedom are 321 since some word completions are not included in the database.

As in Experiment 1, therefore, there were three different word completions for each word stem: the MostProb word completion, characterized by the highest probability (M: 0.33; SD: 0.16); the ImProb word completion, marked by low probability (M: 0.02; SD: 0.01); and a Pseudoword completion. We again created six experimental lists, with lexical variables within a condition matched across lists, such that each participant saw each stem only once and all stems were used with their three completion types the same number of times across participants.

#### 3.1.3 Procedure

Initial paperwork was identical to Experiment 1. Participants then did the word stem completion task; in Experiment 2, the background screen was changed from black to gray for easier viewing. Participants were again instructed to spontaneously generate the first word that came to mind when presented with the three‐letter word stem. However, in this version of the task, generation time was self‐paced, such that participants indicated that they had successfully generated a completion by pressing the space bar. To ensure an adequate time period for collecting cue‐related EEG, the stem remained on the screen for a minimum of 1000 ms even if the participants registered their response within that timeframe. Subsequently, participants were shown a completion from one of the three conditions for 1 s. Different from Experiment 1, participants were asked to make a lexical decision response to the completion (i.e., to indicate with a button press if the presented letter string was a real word). They indicated their response by pressing either the P or Q key on the keyboard, with assignment of yes or no responses to the P or Q key counterbalanced across participants. Finally, participants were asked to type the word they had generated in response to the cue, followed by “enter” to proceed to the next trial, which followed a 1000 ms blank screen.

After the word stem completion task, and following removal of the electrode cap, participants were given a surprise two‐alternative forced‐choice recognition test, probing their memory for all the MostProb and ImProb word completions they had seen (220 trials, divided into two blocks). In this task, participants were instructed to choose one of two words displayed on the screen: either the MostProb or Improb word completion that had been presented in the word stem completion task or a Lure word completion, which they had never seen. Lure words were always the word from the norming data with the second‐highest word probability for that stem. These words were thus less probable than the MostProb completions [95% CI [−0.18, −0.14], $t\left(329\right)=-16.90,p<0.001$] and more probable than the ImProb completions [95% CI [0.13, 0.15], t=38.69,p<0.001]. Participants indicated their choice by pressing either the P or Q key on the keyboard, where each key on the right and left corresponded to two words displayed on the screen. The allocation of correct words to the left or right side was randomly assigned and counterbalanced to ensure an equal 50% distribution on each side. Participants were instructed to make their decisions as quickly and accurately as they could and encouraged to guess when uncertain. To prevent responses that were too hasty and to ensure proper stimulus perception, a mandatory SOA of at least 1 s was implemented.

After completing the recognition task, participants were asked to fill out the vocabulary scale from the Shipley Institute of Living Scale Vocabulary test (Shipley et al. 2009). This scale comprises 40 items, which increase in difficulty. For every item, participants were provided with four answer choices and were tasked with selecting the word that most closely matches the meaning of the given word. Mean raw score on the scale, where each item scores 1 and the maximum possible score is 40, was 31.37 (SD: 3.17).

#### 3.1.4 EEG Recording and Processing

The EEG recording, baselining, and filtering parameters were the same as those used in Experiment 1. Artifact detection and correction procedures followed the same approach as that used in Experiment 1. The mean microvolt thresholds for detecting blinks and eye movements were 87 for the VEOG channel (SD: 40) and 44 for the HEOG channel (SD: 8). For channel drift, the mean microvolt threshold was 128 (SD: 33). On average, approximately 5.0% of trials (range 0.3% to 14.8%) were rejected for the final set of 30 participants.

#### 3.1.5 Event‐Related Potentials

The formation of ERPs and all associated parameters remained consistent with Experiment 1. The match of the completion to participant's expectations was assessed on a trial‐by‐trial basis using the typed responses. If there was an obvious typo (e.g., *burried, where the correct spelling is “buried”), it was manually corrected to correct spelling. However, if the typed response could not be clearly linked to a known word, it was noted as a nonword and excluded from data analysis. Names were considered correctly spelled words even if they started with lower case letters (e.g., annie, where the word stem was “ann___”). Additionally, if the typing response started with a different word stem (e.g., eastern, where the word stem was “est___”) or was replied with “idk” (participants were instructed to type “idk” when they could not come up with any word), both the ERPs to the word stem and the word completion were excluded.

Each item was tagged as a match or mismatch for ERP binning purposes. Mismatch trials were further categorized based on whether there was an inflectional (e.g., “dances” or “danced”) or derivational (e.g., “dancer”) relationship with the presented word completions (in this example, “dance”) of either the MostProb or ImProb conditions. Moreover, these mismatch responses were also categorized by whether they share the same pronunciation (U.S. English) for the initial three letters with that of presented completions (e.g., the pronunciation of d‐a‐n in “danger” /ˈdeɪndʒər/ is different from that in dance /dæns/). Inflectional and derivational forms as well as typical U.S. English pronunciation was referenced from the Oxford English dictionary (Oxford University Press 2024). For Pseudoword trials, pronunciation was determined based on subjective guesses by U.S. English native speakers responsible for sorting. All coding categorizations are available in the Supporting Information.

To further examine the results of the cluster‐based permutation test for Experiment 1, the same approach and statistical thresholds were applied to Experiment 2.

### 3.2 Results

#### 3.2.1 Behavioral Results

Participants were asked to make three responses in the word stem completion task in Experiment 2. First, they signaled that they had successfully generated a completion by pressing the space bar, resulting in an average reaction time of 1694 ms (SD: 1932 ms). Table 5 provides the reaction times corresponding to the five entropy levels. A linear mixed‐effects model with entropy as a continuous fixed effect and participants and stems as random effects was conducted. The model was specified as follows: Stem.Reaction.Time ~ 1 + Entropy + (1|Participant) + (1|Stem). The analysis yielded a significant main effect of entropy on log‐transformed reaction time [b=0.07,SE=0.01,t=5.77,p<0.00 1], revealing increased reaction time as stem entropy increased.

**TABLE 5:** Reaction time of word generation at each stem entropy level.

	LowestEnt (M ± SD)	LowEnt (M ± SD)	MedEnt (M ± SD)	HighEnt (M ± SD)	HighestEnt (M ± SD)
Reaction time	1481 ± 1231	1619 ± 1724	1725 ± 2112	1803 ± 1747	1841 ± 2566

*Note:* The measurement unit for reaction time is milliseconds.

Second, participants completed a lexical decision task upon presentation of the completion. The average reaction time was 636 ms (SD: 655 ms), and the average accuracy rate was 0.98 (SD: 0.15). Table 6 details both the reaction time and accuracy rate for each completion condition. Although there were no significant differences in log‐transformed reaction time among completion conditions $\left[F\left(2,58\right)=1.63,p=0.21\right]$, there were differences in accuracy rate $\left[F\left(2,58\right)=34.44,p<0.001\right]$. Post hoc comparisons using Bonferroni correction indicated significant differences in accuracy rates among all pairs [MostProb>ImProb: 95% CI [0.03, 0.05], $\begin{array}{c} t\left(29\right)=9.00,p<0.001;\text{MostProb}>\text{Pseudo}:95\%\mathrm{CI}\left[0.01,0.03\right], \end{array}$ $\begin{array}{c} t\left(29\right)=5.23,p<0.001;\text{Pseudo}>\text{ImProb}:95\%\mathrm{CI}\left[0.01,0.03\right],t\left(29\right)= \end{array}$ $\begin{array}{c} 3.27,p=0.003] \end{array}$. Nevertheless, the overall mean accuracy was high across all completion conditions, approaching ceiling levels.

**TABLE 6:** Reaction time and accuracy rate of lexical decision task for completion conditions.

	MostProb (M ± SD)	ImProb (M ± SD)	Pseudo (M ± SD)
Reaction time	606 ± 651	636 ± 655	667 ± 660
Accuracy rate	1.00 ± 0.05	0.95 ± 0.21	0.97 ± 0.16

*Note:* The measurement unit for reaction time is milliseconds.

Finally, participants were asked to type the word they had initially generated, and these typing responses were compared with the presented completions to identify match and mismatch trials. This allowed for a comparison of the match rate results with those of Experiment 1. As expected, the match rate for the Pseudo condition was zero. When match rates, with each trial coded as 1 for a match and 0 for a mismatch, were analyzed using a repeated measures ANOVA with three levels of completion condition and five levels of entropy, the general pattern of results was similar to that of Experiment 1. There was a significant main effect of completion condition [$F\left(2,58\right)=409.04,p<0.001$], a significant main effect of entropy [$F\left(4,116\right)=30.53,p<0.001$], and a significant interaction effect [$F\left(8,232\right)=26.46,p<0.001$]. A planned comparison revealed a higher match rate for the MostProb condition (M: 0.28; SD: 0.06) than the ImProb condition (M: 0.07; SD: 0.03) [$95\%\mathrm{CI}\left[0.18,0.23\right],t\left(149\right)=17.57,p<0.001$]. Additionally, as depicted in Table 7 below, entropy affected the match rate for the MostProb (reduced match with higher level of entropy) but not the ImProb condition.

**TABLE 7:** Match rate for completion conditions at each stem entropy level in Experiment 2.

	LowestEnt (M ± SD)	LowEnt (M ± SD)	MedEnt (M ± SD)	HighEnt (M ± SD)	HighestEnt (M ± SD)
MostProb	0.45 ± 0.11	0.29 ± 0.11	0.26 ± 0.11	0.22 ± 0.09	0.17 ± 0.10
ImProb	0.08 ± 0.05	0.06 ± 0.06	0.07 ± 0.06	0.07 ± 0.06	0.07 ± 0.07

Mismatch responses were further categorized according to whether they had an inflectional or derivational relationship with the presented word completions (for the results regarding phonemic categorization, see Appendix B). Among the mismatches, 0.53% were inflectional forms of the presented words, with 0.41% for the MostProb condition and 0.12% for the ImProb condition. Common examples include “fallen” as an inflection of “fall” (MostProb) for the stem “fal___,” or “awake” as an inflection of “awakening” (ImProb) for the stem “awa___.” 1.32% of mismatches were derivational forms of the presented words, with 0.84% for the MostProb condition and 0.48% for the ImProb condition. Examples include “scalar” as a derivation of “scale” (MostProb) for the stem “sca___,” or “scare” as a derivation of “scary” (ImProb) for the same stem. Altogether, only 1.85% of mismatches were morphological variants of (and thus closely related in meaning to) the presented word completions.

At the end of the experiment, participants completed a two‐alternative forced‐choice recognition test. The average accuracy rates for the MostProb and ImProb words were 0.75 (SD: 0.13) and 0.75 (SD: 0.11), respectively. The corresponding average reaction times were 1334 ms (SD: 656) and 1323 ms (SD: 625). Analyses aimed to investigate the effects of expectedness and constraint. For accuracy, a logistic regression analysis was performed using R with the glmer() function from the lme4 package (Bates et al. 2015), with accuracy dummy coded as 1 for correct selection and 0 for incorrect selection (i.e., choosing the lure). For the effect of expectedness, the models for accuracy and reaction time were specified as follows: Accuracy/Reaction Time ~ 1 + Condition + (1|Participant) + (1|Stem). The fixed effect was completion condition (MostProb and ImProb) and the random effects were participants and stems. There was no significant main effect of completion condition for either accuracy $\left[b=-0.03,\mathrm{SE}=0.03,z=-0.85,p=0.40\right]$ or log‐transformed reaction time [b=−0.01,SE=0.01,t=−0.88,p=0.38]. For the effect of constraint, the analysis was limited to those trials with the ImProb completions. The models for accuracy and reaction time were specified as follows: Accuracy/Reaction Time ~ 1 + Entropy + (1|Participant) + (1|Stem). The fixed effect was entropy values as a continuous variable and the random effects included participants and stems. The results revealed no significant entropy effect for accuracy rate $\left[b=0.09,\mathrm{SE}=0.07,z=1.28,p=0.20\right]$ or log‐transformed reaction time $\left[b=0.006,\mathrm{SE}=0.03,t=0.19,p=0.85\right]$.

We also examined performance as a function of match to the participant's initially generated word. Match responses yielded an accuracy of 0.78 (0.14) and an average reaction time of 1380 ms (SD = 804), and mismatches yielded an accuracy of 0.75 (0.11) and average reaction time of 1322 ms (SD = 614). Models were specified as: Accuracy/Reaction Time ~ 1 + Matches + (1|Participant) + (1|Stem). There was a higher accuracy rate for match completions $\left[b=0.09,\mathrm{SE}=0.04,z=2.22,p=0.03\right]$. There was no significant main effect of match responses for log‐transformed reaction time $\left[b=-0.002,\mathrm{SE}=0.02,t=-0.11,p=0.91\right]$.

Therefore, similar to the results for memory task after reading sentences in Hubbard et al. (2019), no effects of expectedness and constraint were found on downstream memory for the words used in the experiment, but participants were more likely to remember words that they had both generated and seen.

#### 3.2.2 ERP Results

##### 3.2.2.1 Word Completions

**FIGURE 7:**
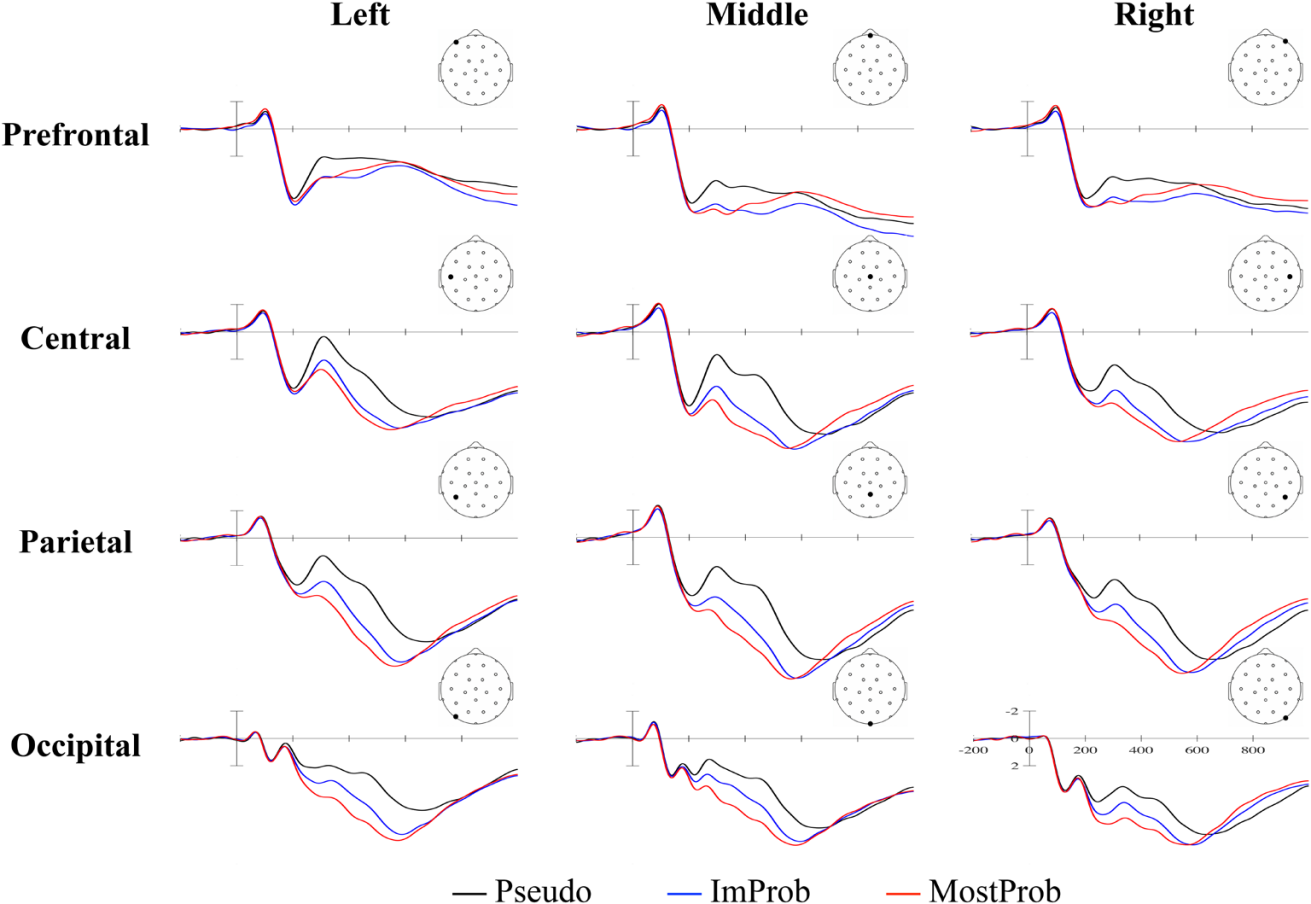
Grand average ERPs at 12 representative channels for the MostProb, ImProb, and Pseudo conditions, time‐locked to presentation of the word completion in Experiment 2.

Figure 7 illustrates the grand average ERPs from 30 participants across the three completion conditions in Experiment 2. Once again, the ERP pattern exhibits typical visual components, followed by an N400 showing a graded response to word probability.

##### 3.2.2.2 N400 Effects (300–500 ms, Central ROI)

**FIGURE 8:**
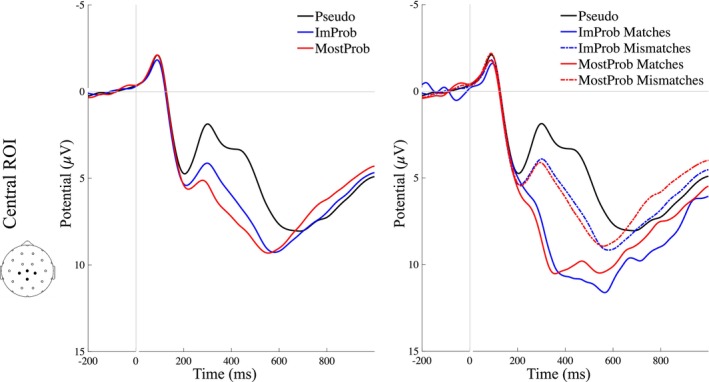
Grand average ERPs over the Central ROI to completion conditions in Experiment 2. The left side of the figure shows the three experimental conditions (MostProb, ImProb, and Pseudo). On the right side of the figure, the MostProb and ImProb conditions are further broken down by whether participants categorized the item as a match or mismatch.

Figure 8 shows ERPs averaged across channels in the Central ROI as a function of condition (left panel) and further binning by match to expectation, here based on participants' typed responses (right panel). Results were very similar to those in Experiment 1. Planned comparisons showed that N400s were reduced for the MostProb condition (M: 7.18 μV; SD: 3.64; SE: 0.66) compared to the ImProb condition (M: 5.93 μV; SD: 4.03; SE: 0.74) [$95\%\mathrm{CI}\;\left[0.75,1.75\right],t\left(29\right)=5.09,p<0.001$], and for the ImProb condition compared to the Pseudo condition (M: 3.17 μV; SD: 3.66; SE: 0.67) [$95\%\mathrm{CI}\;\left[2.12,3.39\right],\;t\left(29\right)=8.88,p<0.001$]. As in Experiment 1, we also found that the N400 amplitude difference is primarily driven by trials where there was an exact match between the presented words and participants' generated word. A linear mixed‐effects model analysis with same settings of fixed and random effects as in Experiment 1 yielded a significant main effect of match responses $\left[b=1.95,\mathrm{SE}=0.18,t=11.13,p<0.001\right]$, but no significant main effect of condition [b=0.21,SE=0.17,t=1.23,p=.22] and no significant interaction effect [b=−0.03,SE=0.17,t=−0.15,p=0.88].

**FIGURE 9:**
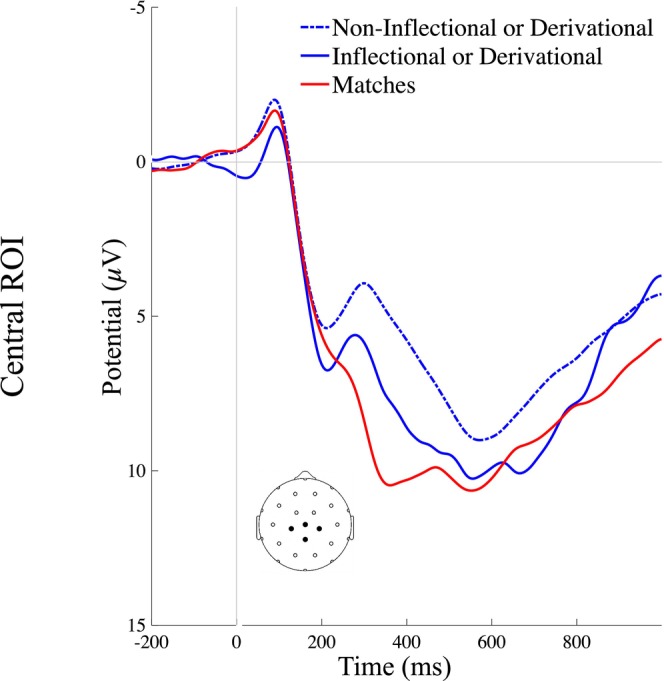
Grand average ERPs over the Central ROI to prediction matches compared to mismatches that shared inflectional or derivational similarity to the presented words and mismatches that did not share this similarity.

Figure 9 shows the Match trials overlapped with Mismatch trials that were binned by whether or not they had an inflectional or derivational relationship with the presented word completions. N400 responses were smaller for Mismatches that shared a root. Given that inflectional/derivational trials comprised only 2.97% of all mismatches, resulting in an imbalance in sample sizes for comparison, a random sampling of non‐inflectional/derivational mismatches was conducted. A *t*‐test was then performed on these samples, comparing the N400 mean amplitudes with those of the inflectional/derivational trials. This procedure was repeated 10,000 times. Across these simulations, 76.40% resulted in a significant outcome (*p* < 0.05), indicating a robust difference in N400 amplitudes between inflectional/derivational mismatches and non‐inflectional/derivational mismatches. Thus, N400 responses are sensitive to partial form and/or meaning similarity even when words do not precisely match expectations. To assess whether lexical properties—particularly frequency differences—might contribute to this effect, given that higher‐frequency words typically elicit reduced N400 amplitudes (Van Petten and Kutas 1990), a similar random sampling analysis was performed using word frequency of the presented word completions as the dependent variable. A significant frequency difference was found in only 31.39% of simulations, and in all such cases, frequency was higher for inflectional/derivational mismatch trials. These results suggest that the reduced N400s to the items that shared a root with the participant's prediction are likely due to that similarity and not to lexical properties like frequency.

##### 3.2.2.3 Anterior Positivity Effects (600–900 ms, Frontal ROI)

**FIGURE 10:**
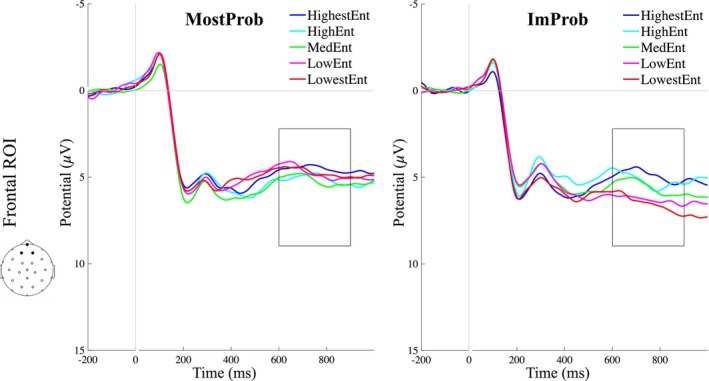
Grand average ERPs over the Frontal ROI (top row) to entropy conditions in Experiment 2. The 600–900 ms time window used to analyze the anterior positivity is marked with a rectangle.

Figure 10 illustrates the pattern across five entropy levels for both the MostProb and ImProb conditions. In contrast to the findings in Experiment 1, there appears to be an enhanced positivity for lower entropy stems in the ImProb condition. Using the same model specification as in Experiment 1, we probed for entropy effects on mean amplitudes in the Frontal ROI within the 600–900 ms timeframe across both the MostProb and ImProb conditions. The results indicated a significant main effect of condition [b=−1.53,SE=0.47,t=−3.23,p<0.01], a significant main effect of entropy [b=−0.32,SE=0.16,t=−1.98,p<0.05], and a significant interaction effect between condition and entropy [b=0.36,SE=0.15,t=2.41,p<0.05].

To examine the interaction, separate linear mixed‐effects analyses were performed for the MostProb and ImProb conditions, with entropy as the fixed effect. The main effect of entropy was found to be significant only for the ImProb condition [b=−0.67,SE=0.24,t=−2.80,p<0.01], indicating increased positivity as the entropy value decreases. Conversely, the effect was not significant in the model for the MostProb condition [b=0.04,SE=0.21,t=0.18,p=0.85]. Thus, different from Experiment 1, in Experiment 2 we found evidence for an anterior positivity effect, elicited to unexpected words and sensitive to prediction strength.

#### 3.2.3 Word Stems

**FIGURE 11:**
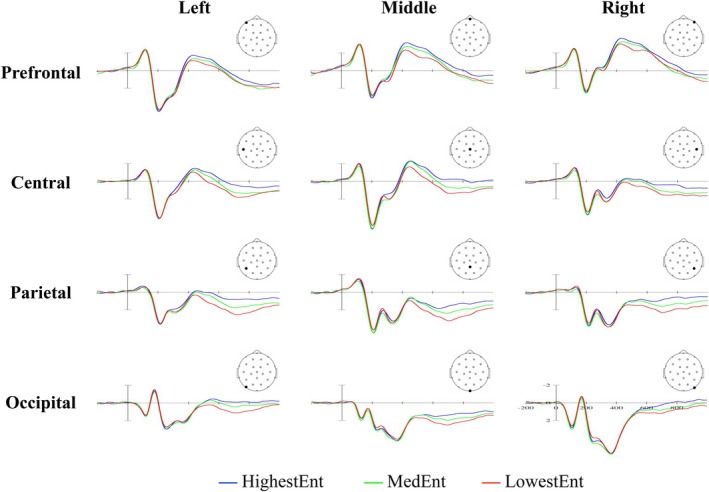
Grand average ERPs at 12 representative channels for three binned entropy levels (highest, medium, lowest), time‐locked to presentation of the word stem in Experiment 2.

Figure 11 presents grand average ERPs time‐locked to the presentation of the word stems. The observed pattern closely aligns with that of Experiment 1. Of particular importance, we again observe a sustained posterior negativity, emerging around 600 ms and showing a graded effect of entropy, with enhanced negativity for higher entropy stems.

##### 3.2.3.1 Centro‐Posterior Negativity (600–850 ms)

**FIGURE 12:**
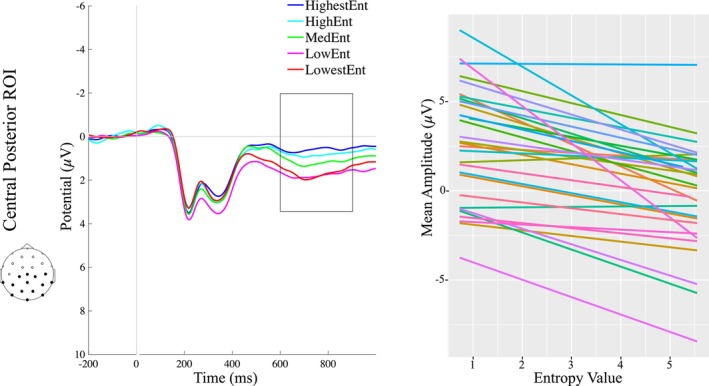
Effects of entropy at the word stem in Experiment 2. The left side of the figure shows grand average ERPs over the central and posterior regions across five binned levels of entropy. The black rectangle shows the critical time window from 600 to 850 ms. The right side of the figure plots the effect of entropy for individual participants.

We first evaluated the effect using the same time frame and ROI (LMCe, RMCe, RDCe, MiCe, MiPa, LLTe, RLTe, LDPa, RDPa, LLOc, RLOc, LMOc, RMOc, and MiOc) identified in the cluster analysis in Experiment 1; see Figure 12. A linear mixed‐effects model with fixed and random effects specified in a manner identical to that in Experiment 1 revealed a significant main effect of entropy [b=−0.59,SE=0.11,t=−5.54,p<0.001]. The right line plot in Figure 12 shows that this effect was again generally consistent across participants.

##### 3.2.3.2 Cluster‐Based Permutation Tests

**FIGURE 13:**
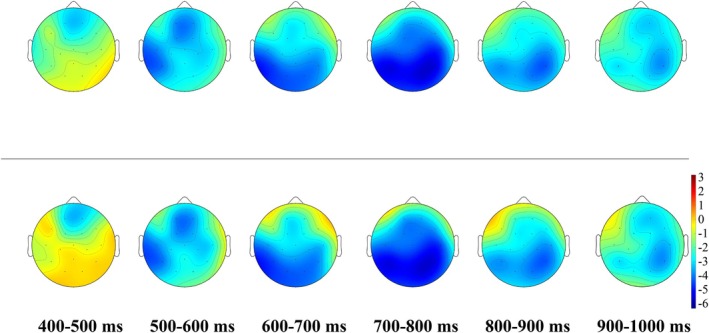
Temporal evolution of the correlation between EEG and entropy. The top row displays topographic plots of the raw results without any masks. The bottom row shows the data masked by the only cluster that passed the threshold of a Monte Carlo *p*‐value less than 0.05. The color bar represents the *t*‐value averaged over the time window.

Although our primary aim was to test the replicability of the effect identified in Experiment 1 using the same channels and time window, we also performed a separate cluster‐based analysis on the data from Experiment 2 to probe the distribution and timing of the effect. There was one cluster with a Monte Carlo *p*‐value less than 0.05 [$t=-39579.42\;\left(\mathrm{sum}\;\text{of}\;t-\text{values in the cluster}\right),p<0.001$], revealing a significant negative correlation between entropy and the EEG signal occurring from 374 to 1000 ms after stimulus onset. Thus, the analyses revealed a similar, albeit more prolonged, timecourse compared to Experiment 1. Figure 13 shows the topography of the cluster between 400 to 1000 ms. The cluster showed a similar posterior maximum as was seen in Experiment 1, but the effect was significant over a broader extent of the scalp. Overall, replicating the findings in Experiment 1, we found a reliable, sustained effect of entropy, maximal over central and posterior regions.

### 3.3 Interim Summary

In line with the outcomes of Experiment 1, the ERP responses to completions showed a graded N400 response associated with word probability, which was primarily driven by N400 facilitations for matches, here binned by participants' typed responses. Further categorization of the typed responses indicated that when they were inflectional or derivational variations (and hence shared the same or similar meanings as the presented words), there was also a decrease in N400 amplitude.

Interestingly, different from Experiment 1, an anterior positivity effect was elicited in Experiment 2, with enhanced anterior positivity to ImProb words following low entropy stems. This result aligns with patterns seen in sentence processing, wherein unexpected words elicit enhanced anterior positivity in strongly constraining contexts. As discussed next, the sensitivity of the anterior positivity to the differing task constraints across Experiment 1 and 2 is revealing of the type of processing that may be driving this effect.

ERPs to the word stems, consistent with Experiment 1, revealed a larger negativity over the central and posterior sites between 600 and 850 ms post‐stimuli as entropy levels increased. This replication, using the same channels and time windows identified in Experiment 1, was further supported by a cluster‐based permutation test, which found a graded effect of entropy that was sustained and broadly distributed over the head, but again maximal over centro‐posterior regions. The consistent findings across the two experiments point to a robust word prediction formation effect influenced by the constraint of the cue (word stem).

Finally, the results of the two‐alternative forced‐choice recognition test indicated a higher accuracy rate for completions generated by participants (match completions), with no further effects of expectedness and constraint.

## 4 General Discussion

The present study aimed to investigate the predictive mechanisms involved in word processing that facilitate rapid comprehension. Previous research has used sentences, manipulating the constraint of the context and then examining ERP responses when people process sentence endings with varying cloze probability, with a focus on the N400 (300–500 ms) and a subsequent positivity over anterior electrode sites (600–900 ms) (Federmeier et al. 2007). These types of studies have shown that N400 amplitudes decrease with higher cloze probabilities, wherein the word being processed is more likely to align with participants' expectations. Unexpected but plausible endings reveal a constraint effect in the form of enhanced anterior positivity for more strongly constraining sentence frames—that is, when participants were likely able to make a prediction, which was then violated.

Although these patterns reveal the impact of prediction on processing, studying the mechanisms of prediction formation is challenging in sentences because those predictions unfold across multiple words. Building on some prior work that has looked at prediction formation within individual words (e.g., based on constraints provided by word‐initial syllables; Roll 2015; Roll et al. 2015; Söderström and Cutler 2023), we here endeavored to study prediction formation using a simpler paradigm based on the kind of word stem completion tasks that have often been employed in the memory literature (Graf and Mandler 1984; Warrington and Weiskrantz 1974). Participants were given a three‐initial‐letter word stem and tasked with providing a completion. This novel approach, which allows us to probe for effects seen in sentences based on constraint and word probability, has the potential to offer new insights into prediction mechanisms. In particular, we can explore the timeframe during which people were presented with a word stem (contextual cue) and needed to generate a word, and thus examine brain responses associated with prediction formation itself.

### 4.1 Cross‐Experiment Summary: Behavior and N400 Effects

Despite differences in the tasks involved, both Experiments 1 and 2 focused on two crucial time windows: the presentation of word stems with varying constraint (operationally defined by entropy) and the presentation of one of three types of word completions: that with the highest word probability for the stem (MostProb), a possible completion with low probability (ImProb), and a pseudoword that fits the stem (Pseudo).

In terms of behavioral responses gathered from participants (self‐reported match/mismatch responses for Experiment 1 and typing responses for Experiment 2), both experiments yielded consistent outcomes. They revealed a higher match rate for the MostProb condition compared to the ImProb condition. There was also a difference in the effect of entropy on word probability, such that in the MostProb condition, the match rate increased as entropy decreased (the cues became more constraining), whereas no significant differences were found among entropy levels for the ImProb condition. These results demonstrate the successful manipulation of completion conditions and entropy levels for the stimuli used in the study.

When analyzing the N400 response to word completions in both experiments, an effect graded by word probability was observed. Similar to findings in sentence contexts, N400 amplitude was most reduced for the MostProb condition, akin to the outcomes seen for expected sentence endings with higher cloze probabilities. Moreover, the experiment design of the present study allowed us to identify on a trial‐by‐trial basis which items matched or mismatched participants' specific expectations, which could be used to sort the N400 responses. Again, the pattern remained consistent across the two experiments, revealing that N400 reductions are primarily driven by cases in which the presented completion matched what the participant expected. A similar pattern has been seen in sentence processing experiments that asked participants to generate predictions for sentence‐ending words and then to judge whether or not the presented completions matched their predictions (Brothers et al. 2015; Lai et al. 2024). Taken together, these results support the idea that graded effects of probability seen on the N400 reflect differing proportions of trials that match comprehenders' in‐the‐moment expectations. N400 facilitations, then, arise when features of a presented word have already been activated through exposure to the context and the expectations that the context afforded.

In addition to asking participants to self‐report whether the presented words matched their expectations (in Experiment 1), in Experiment 2, participants were asked to type the specific words they had generated—a method rarely used in prior research. This approach allowed us to further sort the mismatch trials according to inflectional and derivational relationships with the presented words. These linguistic distinctions are useful for detecting semantic similarity, as inflections share the meaning of the base lexeme, while derivations, although considered separate lexemes, share a similar meaning with the base lexeme (Booij 2006). This categorization thus can serve as one proxy for identifying semantically similar responses. Such responses were rare, accounting for less than 2% of all mismatched responses. Nevertheless, notably, these related mismatches exhibited reduced N400 amplitudes compared to other mismatched responses. This accords with the findings of Lai et al. (2024), who asked participants to self‐rate whether the presented words matched their expectations, shared similar meanings, or were considered unexpected. However, a key limitation of such subjective self‐reports lies in the unclear criteria participants use to evaluate semantic similarity. Here, we were able to demonstrate an effect of similarity using more objective measures. This evidence further supports that N400 amplitudes are sensitive, not just to specific form matches, but more generally to the alignment in semantic features between the predicted and the presented words.

In sentence processing studies, N400 amplitudes are insensitive to constraint when cloze probability is held constant (Kutas and Hillyard 1984). For MostProb words, as for best completions of sentences, constraint and cloze probability are confounded and, as expected, N400 responses decreased as entropy levels decreased in the MostProb condition, consistent with a graded cloze probability effect. More importantly, we examined whether entropy affects the processing of *unexpected* words, where (low) completion probabilities are consistent across levels of entropy. Consistent with the patterns seen in sentence processing, there was no entropy effect for the ImProb condition in either experiment. The N400 patterns align with the behavioral results of participants self‐reporting matches or mismatches, which revealed different match levels as a function of entropy for the MostProb condition, but no entropy effect in the ImProb condition. This supports the notion that context effects on N400 amplitudes are primarily driven by the extent to which an incoming word's semantic features have already been activated and not by the more general state of the semantic system or by activations for other, potentially competing, words (see more extensive review in Federmeier 2022).

### 4.2 Cross‐Experiment Summary: Anterior Positivity

The present study also examined the post‐N400 anterior positivity, an effect that, in sentence processing, has been found to arise for unexpected words when these are encountered in strongly constraining sentences (i.e., where a different word was likely to have been predicted). Interestingly, effect patterns on the anterior positivity were different across the two experiments. In Experiment 1, when participants' task was to judge whether or not the presented completion matched their expectations, there was no evidence of an anterior positivity effect for prediction violations—that is, no evidence of enhanced positivity within the ImProb condition when entropy was lower. However, in Experiment 2, responses over anterior sites in the post‐N400 time window were graded by entropy for the ImProb condition, such that there was enhanced positivity when entropy was lower (and, hence, when prediction was presumably easier and/or stronger).

The difference in effect patterns on the anterior positivity between Experiments 1 and 2 shows that this response is sensitive to the task demands imposed by the lexical decision task compared to the matching task. As such, this pattern may offer valuable insights into the nature of the processes being reflected by the anterior positivity. In Experiment 1, participants only had to indicate if the presented word matched or mismatched their generated completion. If the anterior positivity simply reflected cases in which a prediction was generated and then violated, then we would have expected to observe it in this experimental setting. The fact that we did not suggests that the anterior positivity indexes processes beyond a simple registration of a mismatch with one's prediction. Perhaps critically, in Experiment 1 participants did not need to process the *meaning* of the incoming word in order to note that it mismatched their expectations.

In contrast, Experiment 2 involved a lexical decision task that (likely) required accessing the meaning of the incoming word in order to make a correct response, as the pseudowords were orthographically and phonologically regular and thus differed from the words only in that they did not have a stored meaning. At the same time, participants had to retain their original prediction to be able to produce that word after completing the lexical decision response. Notably, whereas some prior work has hypothesized that the anterior positivity might reflect inhibition of the original prediction in the service of better understanding the sentence (e.g., Ness and Meltzer‐Asscher 2018), the task demands of Experiment 2 required that the generated word be *maintained* across this intervening task, arguing against an inhibition account. Note that in sentence processing, too, it seems likely that when encountering an unexpected word, comprehenders would need to retain at least some of the meaning features associated with the (erroneous) prediction, since those features will often also be linked to the more general context into which the unexpected word is being integrated.

Other accounts have suggested that the anterior positivity might reflect processes involved in revising one's mental model of a sentence in the face of new information from the prediction violation (Brothers et al. 2020; Federmeier et al. 2007). However, different from sentence processing studies, the prediction formed in the current study was based on a very simple context that provided only three letters as word initials. This contradicts the claim made by Brothers et al. (2020) that a late anterior positivity effect requires a rich situation model based on the prior context. In their study, they manipulated and examined the impact of prior context length in three separate experiments. These experiments included minimal contexts, where the probability of a given target word primarily depended on the verb within a single sentence; locally constraining contexts, involving three sentences but with the probability of the target word again primarily determined by the verb in the final sentence; and globally constraining contexts, involving three sentences, where the probability of the target was determined by information built up across all three sentences. The study revealed an enhanced late anterior positivity exclusively for (plausible) unexpected words in globally constraining contexts. Brothers et al. (2020) interpreted the difference in the presence of the anterior positivity across the differing contexts as substantiating the idea that this component functions as an indicator of processes needed to shift or reevaluate a rich mental representation constructed by comprehenders. The view that the anterior positivity is specific to situations in which comprehenders have a rich situation model was already called into question by earlier work showing the positivity in response to atypical exemplars presented after a category cue (e.g., “a kind of tree … sycamore”; Federmeier et al. 2010). However, results from the present study make even clearer that the processes indexed by the late anterior positivity, when encountering prediction disconfirmations, do not necessitate a rich semantic representation of the context.

Instead, the prior literature, in conjunction with the different patterns across Experiments 1 and 2, suggests that the anterior positivity is elicited when participants need to manage the simultaneous activation of two different semantic representations, overcoming one that is strong/dominant to be able to use the other. This may be, in the context of sentence processing, because they need to understand an unexpected word (which may be why anomalous words, which don't fit the message and thus cannot be integrated, do not elicit an anterior positivity; Delong et al. 2011; Kuperberg et al. 2020; Van Petten and Luka 2012) or, in Experiment 2, because they need to make a lexical decision to it. It is interesting that in sentence processing studies too, large anterior positivities to plausible prediction violations are observed in lexical decision tasks, even under conditions in which no anterior positivity is observed during passive comprehension (see discussion in Federmeier 2022). In Experiment 1, where only a match response was required, participants presumably could indicate a mismatch without needing to do anything further with the unexpected word, and thus did not engage the mechanisms underlying the anterior positivity.

What, then, does anterior positivity specifically reflect when the brain needs to “overcome” a strong prediction? One possibility is to think of a strong prediction as being a kind of attractor state in a dynamical system. On this view, as information becomes active, strong constraints allow the formation of what is known as an attractor basin, which enhances positive connections to create a stable representation (Rodd 2020). In order to then process unexpected information, the system needs to move out of this attractor state to be able to activate and maintain semantic information associated with that unexpected input. The anterior positivity may reflect the engagement of neural mechanisms to affect this transition, allowing for a state in which both the original context information and the new input can be active. In sentence processing, this is necessary in order for comprehenders to integrate new information into an established context representation, which would otherwise tend to fall into the attractor dynamics (i.e., the prediction) that the context affords.

However, the formation of an attractor basin does not require a sentential context; attractors can also form based on lexico‐semantic knowledge acquired through learning and experience. Research on the processing of ambiguous words supports the idea that the anterior positivity may be linked to overcoming strong activation states, even when those arise from non‐sentential sources. For example, words like “duck” have a dominant meaning (a noun referring to a bird) and a subordinate meaning (a verb meaning to stoop). In recent work by Chen and Lee (2024), participants were presented with two‐word phrases wherein the first word provided syntactic (but not semantic) information that constrained the part of speech of the second and the second word was either unambiguous, ambiguous and being used in its dominant sense in the phrase, or ambiguous and being used in its subordinate sense. When the first word syntactically constrained the second word to its subordinate meaning, an enhanced anterior positivity was observed compared to other conditions. This finding supports the idea that the anterior positivity indexes mechanisms involved in creating or maintaining initially weaker activation states when there are stronger, competing activations (which, however, cannot simply be inhibited).

This parallels the current study, where the presented words shared the same word stem, and participants' initial word generation likely resulted in a strong activation in the system. In conditions where the word stem had lower entropy (fewer possible words), the preactivated representation may have formed a deeper attractor basin. As a result, processing an unexpected word required mechanisms to break out of this state. This effect was observed specifically in Experiment 2, where task demands required participants to maintain their original completion for the word stem cue through the end of the trial so that they could type it in, but also to engage with the semantics of the input in order to make an accurate lexical decision.

### 4.3 Novel Findings on Prior Word Stem Cues Revealing Entropy Effects on a Late Posterior Negativity

Having established that effects associated with presentation of the completion are similar to those obtained in sentence processing, we can then turn to the primary goal of this study, which was to look for ERP effects associated with the formation of predictions—a process that is difficult to target in sentence processing studies, as prediction formation does not happen at a single, identifiable time point. As we hoped, we were able to detect an effect at the word stem that was sensitive to the ease of prediction formation, as indexed by entropy. In particular, in both experiments, we observed a graded negativity effect within a late time window that commenced by ~600 ms from the onset of the word stem and was largest over centro‐posterior sites. The effects in both experiments manifested an overall similar timing and distribution (although we note that cluster‐based permutation analyses cannot be used to infer precise temporal onsets/offsets or distributional extent; see Sassenhagen and Draschkow 2019). Importantly, then, we find a reproducible pattern linked to the ease of generating a specific word in response to a word stem cue, presumably reflecting either the ease of activating candidates or the ease of selecting one among multiple possible words.

A potential concern with this interpretation is that the word stem completion task explicitly instructs participants to generate candidate words, which may engage active lexical retrieval, search, and selection processes. In contrast, many sentence‐processing studies examine prediction under conditions in which participants are not instructed to anticipate upcoming input, and in some cases are engaged in an unrelated task (e.g., Grisoni et al. 2021). This raises the question of whether the stem‐locked effects observed here reflect predictive processing per se or instead reflect task‐driven retrieval or selection mechanisms. From our perspective, however, this distinction is primarily a functional rather than a mechanistic one. Prediction necessarily entails the activation and retrieval of stored lexical–semantic representations based on contextual constraints. Thus, the processes engaged when participants retrieve and maintain candidate completions for a word stem, particularly under varying degrees of constraint, are not alternative to prediction, but rather constitute one way in which predictive activation is implemented.

At the same time, we acknowledge that the explicit task demands of the present paradigm could modulate how these mechanisms are engaged. Unlike sentence comprehension, where predictive activation emerges spontaneously during message‐level processing, the present task requires deliberate generation and retention of a candidate word. Accordingly, the stem‐locked entropy effects observed here may reflect not only anticipatory activation of a likely upcoming word, but also more explicit effort involved in activating, maintaining, and/or selecting among multiple candidates. Importantly, these demands scale with entropy in principled ways, paralleling findings from sentence‐based prediction studies in which predictability modulates both behavioral and neural responses.

Thus, while the present paradigm does not isolate prediction in a fully naturalistic sense, it provides a controlled means of probing the mechanisms that support anticipatory activation under lexical constraint. The convergence of the present findings with prior sentence‐processing results, including graded N400 effects and task‐dependent anterior positivities, suggests that the word stem completion task engages neural processes that overlap substantially with those involved in prediction during comprehension, even if the task context accentuates explicit generation and maintenance processes. Future work directly comparing explicit and implicit prediction demands within the same paradigm will be important for further clarifying how task goals shape the engagement of these shared mechanisms.

With this interpretation in mind, it is useful to consider how the present stem‐locked negativity relates to prior work examining neural responses to word‐initial cues. As described in the Introduction, prior work in the auditory modality has also identified a response, referred to as the PrAN, that is sensitive to the constraints provided by a word‐initial cue. Whereas initial investigations of the PrAN often compared factorial conditions of accents yielding fewer or more possible completions, a recent reanalysis of Scandinavian language data by Hjortdal et al. (2024) used (cohort) entropy, similar to the current study, and found that PrAN amplitude inversely correlated with entropy. Thus, when comparing the PrAN to the negativity effect found in the current study, a key similarity is that both effects correlate in a graded fashion with the constraint of a sub‐lexical cue. This shared characteristic suggests a similarity across modalities and tasks in the use of word‐initial information to activate sets of word candidates, eliciting neural activity patterns sensitive to constraint and/or entropy. However, beyond this functional similarity, it is difficult to determine if the effects capture shared neural processes, as the patterns differ in timing, distribution, and (with respect to some studies) effect polarity.

PrAN effect timing in previous studies has been variable, but nevertheless notably earlier than the pattern observed in our experiments. However, a timecourse difference might not be surprising even if there were shared mechanisms for the effects, as prior studies have been auditory rather than visual and used a well‐practiced, implicit task (speech recognition). There has also been some variability in the distribution of PrAN‐like effects in the prior literature (mostly left‐biased, but, in the Danish study by Hjortdal et al. (2022), right‐biased), but they have been consistently fairly anterior. A study in Swedish using simultaneous EEG and functional magnetic resonance imaging (fMRI) provided empirical support for a relationship between the PrAN and blood‐oxygen‐level‐dependent (BOLD) activity in the left inferior frontal gyrus (Roll et al. 2017)—a region typically associated with top‐down lexical selection processes (Bedny et al. 2007; Novick et al. 2010; see also León‐Cabrera et al. 2024, for a review of findings supporting the proposed source of the PrAN). In contrast, the effect distribution in the present experiments—even when more broad, as in Experiment 2—has a centro‐posterior maximum.

Perhaps most notably, the majority of studies reporting a PrAN have found an effect pattern with greater negativity when the (time‐locked) phonological cue (e.g., an accent preceding a word's suffix) is more constraining, offering *fewer* possible continuations (Roll 2015; Roll et al. 2017; see review in León‐Cabrera et al. 2024). As reviewed by (León‐Cabrera et al. 2024), the PrAN shares polarity with a broader family of anticipatory negativities, including the sustained semantic prediction potential observed prior to target words in strongly vs. weakly constraining sentences (Grisoni et al. 2017), which León‐Cabrera et al. suggest may indicate a shared preactivation mechanism. In contrast, the negativity observed in the current study shows the opposite pattern, with increased negativity for high‐entropy (less constraining) word stems. Interestingly, however, the only PrAN‐like study run in English, by Söderström and Cutler (2023), also observed a pattern with reversed polarity, where greater negativity was associated with *more* possible continuations. Their target words were all two syllables long, differing only in their initial onset phonemes, which determined either high or low lexical competition. For example, “jealous” begins with [ʤε], which allows 11 times more continuations than [zε] in “zealous.” These (auditory) target words were embedded in carrier sentences, and participants performed a lexical decision task on them. Söderström and Cutler (2023) speculated that the polarity reversal might have arisen because of the different task demands created by the lexical decision task; studies in other languages had generally used a singular/plural or verb tense judgment task. In the present work, however, the polarity of the effect remained constant across the differing task demands of Experiment 1 and Experiment 2 (which, like Söderström and Cutler (2023), involved lexical decisions). Thus, further work is clearly needed to determine what factors influence the direction of PrAN‐like effects and to assess whether there are shared mechanisms at work with the effect we observed here. However, it is notable that across modalities and tasks there is a functionally similar pattern of brain activity graded by the constraint/entropy of a partial word cue.

Considering the literature outside of language, ERP patterns similar to those in the present study—a negative‐going effect (under the same reference settings for the EEG recording), with scalp distribution largest over occipital sites and a sustained temporal pattern—have been observed in studies of visual working memory, on the CDA (contralateral delay activity) component (Vogel and Machizawa 2004; see also Luck and Vogel 2013 for a review). The CDA has been associated with activity important for holding onto representations of visual objects over a delay (i.e., with visual sensory memory). Research has shown that the CDA is sensitive to visual memory capacity, measured by the quantity of objects to be memorized within one of the visual fields. As the number of objects for memorization increases, an enhanced, sustained negativity is observed over posterior sites contralateral to the stimuli. In the current study, we also observed an enhanced negativity, exhibiting a correlation with entropy. If the response we observe is related to the CDA, this might suggest that participants initially activate and retain multiple potential word completions to a given stem, possibly until one of them can be selected from the set. Of course, again, further investigations are needed to ascertain whether this negativity might be functionally related to that observed in visual working memory studies.

The current study takes a novel approach by using a word stem completion task, which allows precise manipulation and control of cue constraints, to probe the pre‐target time window and investigate prediction formation. As noted in the Introduction, explicitly cueing participants to produce a completion from a given word stem differs in some respects from prediction formation in sentence comprehension. Nevertheless, as supported by prior research on the role of production in predictive mechanisms (Dell and Chang 2014; Federmeier 2007; Pickering and Gambi 2018; Pickering and Garrod 2013), the word production process in this paradigm, where orthographic and phonological cues from the stem guide word generation, offers valuable insight into how constraints influence prediction. Irrespective of its exact nature or similarity to other known ERP responses, the pattern observed in the present study highlights the effect of constraint/entropy on prediction‐related processes. The results imply that, in response to a predictive cue, comprehenders activate information and that the amount of information activated and/or the effort involved in activating and/or selecting among that information is sensitive to the distribution of possible outcomes linked to that cue. Such a readiness presumably supports prediction and the efficient word processing it permits.

## 5 Conclusion

Although the word stem completion paradigm is different in important ways from natural language comprehension, the ERP results, particularly the N400 and late anterior positivity patterns, indicate that word processing in this paradigm engages processes that are comparable to those studied in sentence processing. It seems that, to the brain, a cue consisting of a three‐initial trigram and a prior context conveyed through a sentence frame share important commonalities in that both can yield the pre‐activation of words and their meanings based on knowledge stored in long‐term memory. Both also yield variability in the extent to which they narrow down the set of candidate words, which we here measured as the entropy of the word stem. In both cases, when people are then presented with a word that mismatches their predictions, they elicit a larger N400 and, in some cases, a subsequent anterior positivity. Here, we saw that the positivity was sensitive to task demands, appearing only in Experiment 2 when participants needed to set aside (but maintain) their original prediction to make a lexical decision judgment, before then reporting what that original prediction had been. In future work, it will be important to further compare sentence and single‐word processing to uncover the commonalities and differences in how the cues available from word stems and sentence contexts serve to afford prediction and facilitate word processing.

Importantly, we found an effect of entropy at the presentation of the word stem itself, manifested through an enhanced late posterior negativity. This, then, reveals that the brain is using available information to preactivate in a manner that is sensitive to the predictive strength and scope of the cue, measured as those predictions are being formed, rather than just through inference based on downstream match or mismatch effects. Future work can build on this novel measure to probe the factors that promote and shape prediction formation. Overall, these findings thus shed light on the shared mechanisms between sentences and single‐word processing, which allow comprehenders to rapidly pre‐activate information about likely upcoming words and to use that information in task appropriate ways when they then encounter words that are or are not consistent with their predictions.

## Author Contributions

**Hui‐Sun Chiu:** conceptualization, writing – original draft, writing – review and editing, data curation, formal analysis, project administration, visualization, methodology, investigation, validation. **Ryan J. Hubbard:** methodology, software, conceptualization, writing – review and editing, supervision, validation, project administration, data curation, investigation. **Kara D. Federmeier:** conceptualization, methodology, funding acquisition, resources, supervision, writing – review and editing, investigation, project administration.

## Funding

This work was supported by National Institutes of Health, R01AG026308.

## Conflicts of Interest

The authors declare no conflicts of interest.

## Supporting information


**Data S1:** Hui‐sun_Psychophysiology_paper_Supporting Information.

## Figures and Tables

**FIGURE B1:**
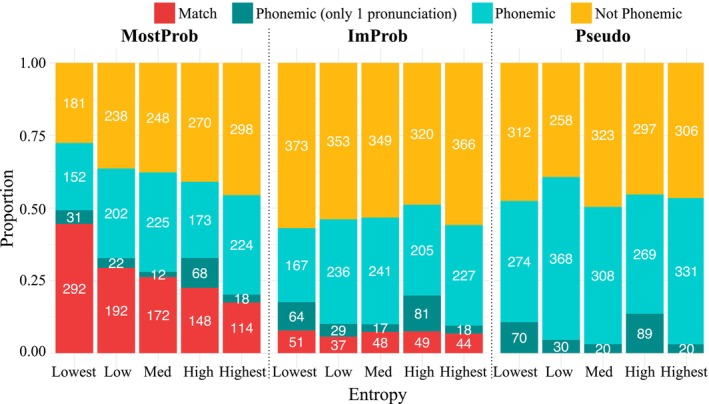
A proportional breakdown of matches and every phonemic categories in Experiment 2. The numbers labeled in white represent the total number of trials of each proportion.

**FIGURE B2:**
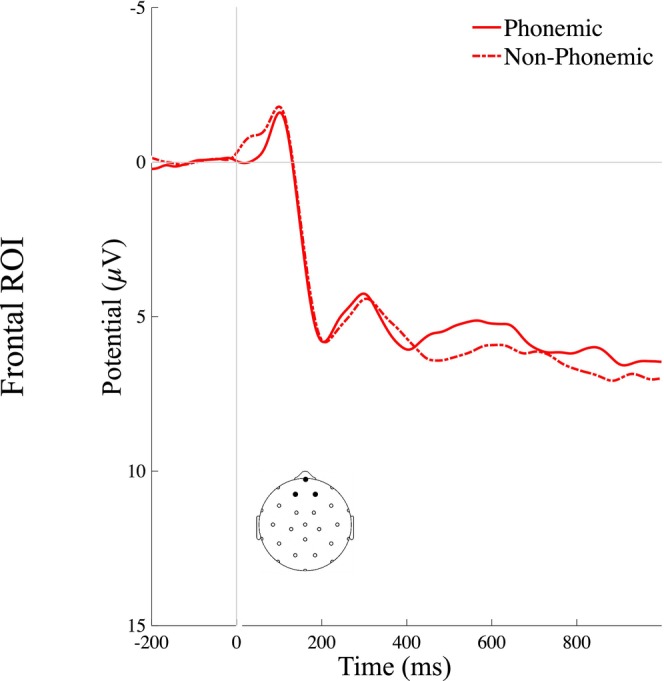
Grand average ERPs at frontal ROI to phonemic conditions (including ImProb trials with lowestent and lowent entropy stems).

## Data Availability

The data that support the findings of this study are available from the corresponding author upon reasonable request.

## Endnotes

^1^
Approximately 24.55% of the 330 stems could independently function as lexicalized words in standard dictionary entries or everyday speech (e.g., act, bus, and cat). However, based on responses collected during the norming stage and the main experiment, instances in which responses treated the stems as standalone lexical items (e.g., acted, buses, and cats) were very rare, accounting for approximately 1.3% of all trials across all participants in the main experiment.

## Appendix A Word Stem and Completion Stimuli

A complete list of materials is provided below, including 330 word stems and their completions across three conditions for Experiment 1 and Experiment 2 (with only the ImProb condition differing between the two experiments)

**Table:**

Stem	Completion			
	MostProb	ImProb		Pseudo
		Experiment 1	Experiment 2	
abo___	about	abolition	above	aboods
abs___	absolute	absurd	absurd	abstest
acc___	account	access	access	accuts
act___	action	activity	actual	actir
adm___	admit	administrative	admiral	admid
adv___	advance	advantages	advantage	advorts
aff___	affect	affirmative	affront	affab
ali___	align	alias	alike	alimid
all___	allow	allergy	alliance	allind
amo___	amount	amorphous	amortize	amoode
ana___	anagram	anatomy	anatomy	anapredas
ang___	angle	angus	angus	angsa
ann___	annotate	annihilate	announce	annat
app___	apple	appointment	appear	approofs
arm___	army	arming	armpit	armfut
art___	artist	arthur	article	arthorm
ass___	assist	assemble	assure	assait
att___	attention	attraction	attorney	attont
aut___	autumn	authentic	authentic	autope
awa___	away	awakening	awakening	awator
bal___	balance	balmy	bald	balla
ban___	band	banquet	bang	bandal
bas___	bass	baste	basin	basps
bat___	battle	baton	baton	batil
bea___	beach	beam	beam	beases
bee___	been	beekeeper	beep	beethe
bel___	bell	belief	below	belrius
ben___	bent	benny	bengal	bendlem
bla___	black	blanket	blame	blatise
bli___	blind	blizzard	blizzard	blilks
blo___	blow	blouse	block	bloy
bon___	bonfire	bonanza	bonus	bonepi
bot___	bottom	botched	botch	botak
bou___	bounty	bourbon	bound	bourny
bra___	brain	brazil	brass	braits
bre___	break	breed	breach	brears
bri___	bright	brian	bridge	briot
bro___	brother	bronze	broad	brom
bur___	burn	burst	burger	burbam
bus___	busy	bustle	bush	bushop
cal___	call	calamity	calendar	calkore
cam___	camera	camouflage	cameo	camin
can___	candle	cans	cancer	cannien
cap___	capital	captured	capable	capstend
car___	cart	carbon	carbon	cargoot
cat___	catch	catholic	catholic	catatax
cau___	caution	cauter	caught	cauns
cel___	cell	celestial	celebrate	cellior
cen___	century	centennial	centennial	centipe
cha___	change	charge	chance	charmel
che___	cheese	checkers	chest	chessar
chi___	chicago	chimp	china	chirk
cho___	chocolate	chorus	choice	choss
cit___	city	citrus	citizen	cition
cla___	clap	clasp	claw	clard
cle___	clean	clench	clench	cleadased
clo___	clown	clot	clock	clons
clu___	clue	cluck	clumsy	clufo
coa___	coal	coaster	coast	coadeet
col___	cold	collar	collar	collipe
com___	computer	comfortable	company	commene
con___	continue	convince	consider	condect
coo___	cool	cooperate	coop	cood
cop___	copper	copious	copious	copces
cor___	cord	cork	core	corrive
cou___	couple	courier	court	coud
cra___	crazy	crawl	crack	crars
cre___	create	crew	crew	crepects
cri___	crime	cries	cricket	crifts
cro___	crow	crotch	crop	crongs
cru___	crush	crumbs	cruel	crun
cur___	curious	curfew	curfew	curgs
dan___	dance	dandruff	dandruff	dangriff
dar___	dark	darn	darn	dars
dea___	deal	dean	dean	dease
deb___	debt	debilitating	debris	debarca
dec___	december	declare	decent	declite
def___	defense	deficient	defeat	defnin
deg___	degree	degenerate	degrease	deglies
dem___	democrat	demolition	demand	demops
den___	dent	denial	deny	denmern
dep___	depend	deprived	deputy	depites
des___	destiny	deserve	deserve	descrey
det___	deter	detonate	detail	detoos
dev___	develop	devise	device	devarm
dia___	diamond	dialogue	dialogue	dianives
dir___	direct	dire	dire	dirmbive
dis___	dislike	disease	disease	dispopt
div___	divide	divert	divorce	dival
doc___	doctor	docile	docket	doctrent
dou___	doubt	dour	dour	dourn
dra___	drama	drastic	draw	drace
dre___	dread	dregs	drew	drell
dri___	drink	drift	drift	drig
dro___	drop	drowsy	drove	drolf
ear___	early	earnest	earlobe	earbah
eas___	easy	eastern	eastern	eash
eff___	effect	effeminate	effeminate	effond
ele___	element	elevator	elevator	elecrite
emp___	empire	employment	emporium	empatrore
eng___	english	engage	engage	engate
ent___	enter	entity	entire	entle
equ___	equal	equipment	equipment	equactent
est___	estimate	esther	esteem	estailend
eve___	evening	eventually	eventually	evene
exa___	exam	exaggerate	exalt	exarbler
exc___	excite	excess	excess	excullate
exe___	execute	exemplar	exemplar	exectrir
exp___	experience	expensive	expensive	explorn
ext___	extent	exterminate	extreme	extire
fac___	face	faction	faction	facorm
fai___	faith	fairly	fairly	faifs
fal___	fall	fallow	falsify	falsh
fam___	family	familiar	famous	famear
fas___	fast	fascinate	fascinate	fashoed
fat___	father	fats	fatal	fattop
fea___	feather	fealty	fealty	feakness
fee___	feel	fees	feeble	feep
fel___	felt	felicity	felicity	felat
fig___	figure	fight	fight	figote
fil___	filter	filthy	file	filns
fin___	finish	finger	finger	finams
fir___	first	firs	firs	firtty
fla___	flag	flare	flash	flanur
fle___	flea	flesh	flesh	fleps
fli___	flip	flirt	flit	flitry
flo___	flower	float	floor	flopel
flu___	fluid	fluffy	fluffy	flunt
fol___	follow	folks	folk	folf
foo___	food	football	foot	foom
for___	form	forgive	forget	forbate
fou___	found	fought	fought	fous
fra___	frank	franchise	frantic	fraze
fre___	freedom	freak	fresh	fredgy
fri___	friday	friar	friend	frieves
fro___	from	frogs	froze	frobin
fun___	funny	function	funeral	funna
fur___	furry	furs	furious	furlale
gal___	gallon	gallows	gallery	gallond
gar___	garage	gary	garlic	garges
gen___	general	genetic	genius	genna
gla___	glad	glades	glare	glame
gra___	grape	grand	grand	grase
gre___	great	gray	gray	greaker
gro___	group	groom	groom	groober
hal___	hall	hallucination	half	halofid
han___	hand	hanker	hang	hantrile
har___	hard	hardware	harbor	harpar
hea___	health	heavy	heaven	heara
her___	hero	herald	herd	herbebs
hol___	hold	holler	holler	holses
hon___	honey	hone	hone	honas
hor___	horror	horizon	horn	horch
hos___	hostile	hostess	hostel	hossets
hun___	hungry	hunch	hung	hune
hur___	hurry	hurling	hurray	hurn
ide___	idea	identify	identify	ideens
imp___	imply	impossible	impress	impobe
inc___	include	incense	inch	incule
ind___	indian	indeed	indeed	indeit
inf___	infinite	infirmary	influence	infettink
ins___	insurance	instrument	instead	insheed
int___	interest	introduce	introduce	intofes
inv___	invite	involve	involve	invame
kin___	kind	kinsmen	kink	kint
lan___	land	lance	lance	landa
lat___	later	latte	latrine	latet
lea___	leaf	league	least	leams
leg___	legs	legacy	legal	legorn
lib___	library	liberation	libel	libit
lim___	lime	limo	limo	limna
lit___	little	liter	liter	litipins
loa___	load	loathing	loam	loap
loc___	lock	locust	locust	locots
loo___	look	loose	loose	lools
mag___	magazine	magnitude	magnitude	maghem
mai___	main	maintain	maiden	maist
mal___	mall	malignant	malign	malyan
man___	manhole	maniac	maniac	mangien
mar___	march	marvelous	market	marps
mas___	master	masquerade	mash	masmit
mat___	matter	material	material	matshest
mea___	meat	measly	meadow	meadue
med___	medical	medal	media	medot
men___	mental	menace	menu	mentnear
mer___	mermaid	mercer	merit	mermern
met___	metal	meta	metric	methan
mil___	million	mildew	mile	miltariue
min___	minute	minor	mind	minid
mis___	mistake	miserable	mislead	mistops
mod___	model	module	module	modose
mon___	monday	monitor	month	mongion
mor___	more	mormon	moron	morsts
mot___	mother	motive	motion	motah
mou___	mouse	mourn	mourn	mountaste
mul___	mulch	mulligan	mulligan	mulca
mus___	music	mush	must	mushlat
nat___	nature	nativity	native	nathod
not___	nothing	notify	notify	noths
off___	office	offensive	offense	offot
ope___	open	operate	opera	opecent
out___	outside	outfit	outfit	outhort
pac___	pack	pacifier	pacifier	pach
pai___	pain	pairs	pair	paids
pal___	palace	palsy	palsy	palk
pan___	pancake	panther	panel	panso
par___	park	paradise	parent	parareans
pas___	pass	paste	pasta	pasteen
pat___	pattern	patient	patient	pateines
pen___	pencil	penetrate	pension	penfol
per___	person	perform	period	persant
pic___	picture	pickle	pickle	pice
pil___	pillow	piledriver	pilot	pilses
pin___	pint	pinkie	ping	pinlave
pla___	place	plan	plan	plapes
ple___	please	plenty	plenty	plem
plu___	plural	plump	plug	plusel
pol___	police	polite	polite	polose
pop___	popcorn	pops	population	pophort
por___	porch	portrait	portal	porrepts
pos___	position	possess	pose	poss
pot___	potato	potential	potential	potise
pra___	pray	prairie	prairie	praws
pre___	present	pregnant	pregnant	preulged
pri___	price	privilege	print	pristond
pro___	produce	probably	proof	propean
pur___	pure	pursue	pursue	purst
qua___	quality	quantum	quarter	qualliny
que___	question	queasy	queer	quens
qui___	quick	quiz	quiz	quims
rad___	radical	radiology	radius	rados
rai___	rain	raise	raise	rais
ran___	random	rang	range	rantuily
rat___	rattle	ratio	ratio	ratten
rea___	real	rear	ready	rearl
rec___	record	recall	recent	receine
red___	redo	reduce	reduce	redeap
ref___	referee	refuse	refuse	refach
reg___	regular	regret	regret	regalmy
rel___	relish	relax	relax	reloctons
rem___	remember	remodel	remain	remyles
rep___	represent	replica	replica	repoit
res___	rest	result	result	resoct
ret___	return	retrieve	retain	retle
rev___	reverend	revenge	revenge	revetens
roo___	roof	roost	rookie	rooch
rou___	round	roulette	roulette	routh
sal___	salt	saloon	salon	salpo
sca___	scale	scarce	scary	scambs
sch___	school	scholarship	scholar	schematly
sco___	scold	scott	scotch	scowd
scr___	scream	scrooge	screech	scrock
sea___	seal	seas	season	seafoup
sec___	second	secede	security	securd
see___	seen	seeps	seep	seenak
sel___	seldom	sellout	selfie	selsim
sen___	senator	sensitive	senior	senave
ser___	service	series	series	serd
sev___	severe	severity	severity	sevate
sha___	shame	shall	shall	shalop
she___	sheep	sheen	sheriff	shecs
shi___	ship	shingle	shield	shilab
sho___	show	shoot	shoot	shogs
sig___	signature	sight	sight	sigli
sil___	silent	sill	silicon	silice
ski___	skip	skinny	skirt	skiets
sla___	slave	slaughter	slap	slals
sli___	slice	slit	slim	slimp
slo___	slow	slogan	slogan	slofty
smi___	smile	smitten	smitten	smillen
smo___	smoke	smock	smock	smoofs
sna___	snap	snag	snatch	snage
sno___	snow	snob	snoop	snop
sol___	sold	solemn	sole	solinits
sor___	sorry	sorcery	sordid	sorsel
sou___	soul	source	source	soungert
spe___	special	species	spent	spelk
spi___	spice	spite	spirit	spinyll
spo___	spoon	spontaneous	sponge	spolt
spr___	spring	spray	spread	spreet
sta___	stand	stare	staff	stard
ste___	stem	stereo	stew	steis
sti___	still	stitch	stitch	stinds
sto___	stop	stolen	stone	stoem
str___	strength	strip	strike	strelles
stu___	student	studio	stuck	studar
sub___	subway	submit	submit	subsor
sup___	supper	supreme	supreme	suppint
sur___	surprise	surplus	surface	surdaws
sus___	sustain	sustenance	suspicion	susku
swe___	sweet	swell	swell	sweline
swi___	swim	swine	switch	swize
sym___	symbol	symphony	symphony	symnal
tal___	tall	talc	tale	talt
tea___	team	teak	teats	teanop
tel___	telephone	telescope	telescope	televill
tem___	temper	temptation	template	temes
ten___	tent	tentacles	tension	tengal
ter___	terrace	terrific	terrific	terbe
tha___	that	thaw	than	thance
the___	theme	therapy	therapy	thewed
thi___	this	thirst	thirst	thisk
tho___	thought	thomas	thorn	thol
thr___	three	thrash	throat	thraft
tin___	tint	tins	tingle	tinfark
ton___	tone	tony	tonsil	tonance
tou___	touch	tourist	tough	tound
tra___	train	transfer	travel	trass
tre___	tree	tremendous	trek	trecanious
tri___	trip	tried	trick	trifern
tru___	truth	trunk	trunk	trums
twe___	tweet	tweezers	tweezers	twerth
uni___	unicorn	united	unique	unicrix
unl___	unleash	unlisted	unlucky	unloque
val___	value	valley	valley	valat
var___	various	variation	varnish	vareen
ver___	very	versus	versus	verg
vic___	victory	vicar	vicar	victaw
vir___	virtue	virgil	virulent	virit
vis___	vision	vise	visa	vislaes
war___	warrior	warden	warden	warcere
was___	wash	wastrel	wastrel	wask
wea___	weather	weakling	weak	wealod
wel___	welcome	welsh	welfare	welft
whe___	when	whelp	whelp	wheek
wil___	will	wily	wily	wilgel
win___	window	wins	wine	wint
wis___	wisdom	wisp	wisp	wisbem
wor___	word	worm	worse	worps
you___	your	youthful	youthful	yoursope

## Appendix B Results of Phonemic Categorization

For the phonemic categorization of the typed responses in Experiment 2, which includes all mismatches, including the Pseudo trials, 48.27% of responses shared the same initial pronunciation for the word stem, while the remaining 51.73% began with different pronunciations. In some cases there was only one possible pronunciation for the word stem, as seen in examples like “loa___”, “men___”, “scr___”, “fee___”, and “sym___,” etc. These made up 14.05% of all phonemically similar responses (6.78% of mismatches). The figure below presents a stacked bar chart illustrating the proportions of matches and each phonemic category across all trials for each condition and all participants (Figure B1).

Approximately half of the responses shared the same phonemic pronunciation for the initial three letters, while the other half did not. We specifically analyzed the ImProb completions with strongly constraining word stems, including both the LowestEnt and LowEnt conditions. The comparison focused on phonemic versus non‐phonemic completions, excluding phonemic responses where only one possible pronunciation of the word stem was identified across all participant‐generated responses. We expected to observe enhanced anterior positivity for the non‐phonemic category, as these completions were expected to more strongly disconfirm the words generated by participants. While the figure below appears to show the anticipated pattern (Figure B2), the results were not statistically significant. A linear mixed‐effects model analysis was conducted with phonemic categorization as a fixed effect and participants and word stems as random effects for the ImProb completions with LowestEnt and LowEnt word stems. The analysis revealed no significant main effect of phonemic status [b=−0.66,SE=0.65,t=−1.02,p=0.31].
